# Transcriptomic Analysis Reveals Novel Mechanistic Insight into Murine Biological Responses to Multi-Walled Carbon Nanotubes in Lungs and Cultured Lung Epithelial Cells 

**DOI:** 10.1371/journal.pone.0080452

**Published:** 2013-11-19

**Authors:** Sarah Søs Poulsen, Nicklas R. Jacobsen, Sarah Labib, Dongmei Wu, Mainul Husain, Andrew Williams, Jesper P. Bøgelund, Ole Andersen, Carsten Købler, Kristian Mølhave, Zdenka O. Kyjovska, Anne T. Saber, Håkan Wallin, Carole L. Yauk, Ulla Vogel, Sabina Halappanavar

**Affiliations:** 1 National Research Centre for the Working Environment, Copenhagen, Denmark; 2 Environmental and Radiation Health Sciences Directorate, Health Canada, Ottawa, Ontario, Canada; 3 Danish Technological Institute, Taastrup, Taastrup, Denmark; 4 Department of Science, Systems and Models, Roskilde University, Roskilde, Denmark; 5 Department of Micro- and Nanotechnology, Technical University of Denmark, Kgs. Lyngby, Denmark; 6 Department of Public Health, University of Copenhagen, Copenhagen, Denmark; Northwestern University Feinberg School of Medicine, United States of America

## Abstract

There is great interest in substituting animal work with *in vitro* experimentation in human health risk assessment; however, there are only few comparisons of *in vitro* and *in vivo* biological responses to engineered nanomaterials. We used high-content genomics tools to compare *in vivo* pulmonary responses of multiwalled carbon nanotubes (MWCNT) to those *in vitro* in cultured lung epithelial cells (FE1) at the global transcriptomic level. Primary size, surface area and other properties of MWCNT- XNRI -7 (Mitsui7) were characterized using DLS, SEM and TEM. Mice were exposed *via* a single intratracheal instillation to 18, 54, or 162 μg of Mitsui7/mouse. FE1 cells were incubated with 12.5, 25 and 100 μg/ml of Mitsui7. Tissue and cell samples were collected at 24 hours post-exposure. DNA microarrays were employed to establish mechanistic differences and similarities between the two models. Microarray results were confirmed using gene-specific RT-qPCR. Bronchoalveolar lavage (BAL) fluid was assessed for indications of inflammation *in vivo*. A strong dose-dependent activation of acute phase and inflammation response was observed in mouse lungs reflective mainly of an inflammatory response as observed in BAL. *In vitro*, a wide variety of core cellular functions were affected including transcription, cell cycle, and cellular growth and proliferation. Oxidative stress, fibrosis and inflammation processes were altered in both models. Although there were similarities observed between the two models at the pathway-level, the specific genes altered under these pathways were different, suggesting that the underlying mechanisms of responses are different in cells in culture and the lung tissue. Our results suggest that careful consideration should be given in selecting relevant endpoints when substituting animal with *in vitro* testing.

## Introduction

It is anticipated that the widespread use of engineered nanomaterials (ENM) in consumer products will lead to increased human exposure as a result of environmental contamination and using consumer goods containing ENM. Because of their nano size and distinct physico-chemical properties, exposure to ENM is suggested to cause different toxic responses than exposure to their larger counterparts. However, hazard characterization and human health risk assessment of ENM is hampered by a lack of validated toxicological methods for assessing ENM, and a lack of appropriate dose metrics and methodologies for sample preparation (reviewed in 1). Indeed, it has been estimated that it would require billions of dollars and close to 50 years to employ traditional toxicological methods to assess the ENM currently on the market [2]. Thus, there is an urgent need for the development of rapid alternative testing strategies. 

Many efforts have been made to develop inexpensive, rapid, and simple *in vitro* screening assays to assess and predict ENM-induced toxicity. Although limited in number, studies comparing *in vitro* and *in vivo* biological responses following ENM exposure have found generally poor concordance between the two test systems [3,4]; these experiments question the relevance of *in vitro* findings to *in vivo* health effects for ENM. For example, Seagrave et al. [3] found that diesel exhaust particle extracts were less toxic in *in vitro* tests in rat alveolar macrophages, but more toxic in rat lungs [3]. Sayes et al. [4] reported little correlation in the toxicological properties of five different types of fine- and nano-particles following *in vivo* instillation of rats compared to *in vitro* exposures using three different cell culture models (rat lung epithelial cells, rat primary alveolar macrophages and co-culture of lung epithelial and primary alveolar macrophages) [4]. Broadly different responses were also reported for *in vivo* compared to *in vitro* exposures to fine zinc oxide particles, and to fullerene, carbon nanotubes (CNT), gold and silver nanoparticles [5,6], reviewed in 7,8. Differences in *in vitro* and *in vivo* responses may be expected because *in vivo* studies evaluate toxicity in whole organisms or organs with complicated interplay between multiple cell types, whereas *in vitro* studies primarily focus on understanding the response of a single cell type isolated from a specific organ. It should also be noted that the comparisons described above are limited to a small set of biological endpoints that include inflammatory markers, oxidative stress, cytotoxicity and markers of tissue damage. 

In contrast to the studies described above, there appears to be agreement between the *in vitro* and *in vivo* models for the evaluation of genotoxic potential of nanomaterials (reviewed in 9). These findings suggest that careful selection of cell types, toxicity endpoints and knowledge of the related tissue physiology is important to derive meaningful comparisons. Moreover, the data demonstrate that the biological responses and pathologies associated with exposure to ENM are complex, and involve perturbations of several pathways and functions. The use of systems biology approaches to gain knowledge of the intricate relationship between the pathways leading to toxicity in these systems is required not only to understand the underlying etiologies of ENM-induced effects, but also to validate the relevance of *in vitro* results, undertaken to predict *in vivo* risk. 

CNTs are cylindrical carbon allotropes. Their nano size, high aspect ratio (length/width), and fibre shape render their properties asbestos-like resulting in potentially higher toxicity than larger sized particles [10-12]. CNTs are categorized as either single-walled (SWCNT, single sheet of graphene) or multi-walled (MWCNT, multiple sheets of graphene) based on their wall numbers. MWCNT, which are the focus of the current study, are widely used in many industrial and biomedical applications [11,13,14] and consequently, occupational exposure to MWCNT has increased. *In vivo* animal exposure models clearly demonstrate that inhalation or instillation of MWCNT into the lungs induces inflammation, persistent interstitial fibrosis, and granuloma formation in rodents [15-20]. Studies on mice given intraperitoneal injections of MWCNT of different shapes and lengths into the mesothelial lining of the peritoneal cavity show that length and diameter are important for the infiltration of inflammatory cells into the lungs and lung fluid [21,22] inciting inflammation. MWCNT are biopersistent, cause chronic tissue injury and at least in some situations, are carcinogenic. Indeed, it was recently shown that p53 heterozygous mice or rats given intraperitoneal or intrascrotal injection of MWCNT develop mesotheliomas in a dose-dependent manner [23,24] (NIOSH bulletin). For these reasons, we believe that at the doses tested, MWCNT should be considered as causing serious health effects and possibly being carcinogenic. 

The primary objective of the present study was to delineate the mechanisms underlying the biological responses of an *in vitro* lung epithelial monolayer cell culture model, relevant to the *in vivo* lung tissue response, following exposure to MWCNT to shed light on the utility of *in vitro* models to predict adversity *in vivo*. MWCNT- XNRI -7 (Mitsui7) was used as a model MWCNT particle. To accomplish our objective, we characterized global transcriptional responses in the lungs of C57BL/6 mice exposed to Mitsui7 and compared them to transcriptional profiles from mouse lung epithelial cells exposed to Mitsui7 in culture. C57BL/6 mice were used to study *in vivo* effects because all of our previous and ongoing *in vivo* studies on different particle types have been conducted on this strain. Therefore, the results of this study will also enable consistent and systematic comparisons of biological responses across particle types. The mouse lung epithelial cell line FE1 was used to investigate *in vitro* responses to Mitsui7. FE1 is a spontaneously immortalized lung epithelial cell line derived from a normal healthy Muta^TM^Mouse. This cell line retains key endogenous metabolic capacity and p53 signalling, and presents both a type I and type II alveolar phenotype. Because the Muta™Mouse model contains a *lacZ* mutation reporter transgene, this cell line also enables an assessment of the mutagenic effects of nanomaterials and has been extensively used in our laboratory for studying the genotoxicity of chemicals and nanomaterials [25-31]. 

*In vitro* doses were chosen based on their relevance to *in vivo* lung burden and also to reflect the doses reported in the literature. DNA microarrays were used to examine the responsive biological pathways and functions altered in FE1 cells incubated in the presence of 12.5, 25, 100 µg/ml Mitsui7 relative to lung tissues exposed via intratracheal instillation to 18, 54, 162 µg/mouse Mitsui7. Samples were collected at 24 hours post-exposure. Statistical and systems biology tools were employed to provide a global foot-print of Mitsui7-responsive pulmonary transcriptome in the two widely studied models. *In vivo* characterization of lung inflammation and validation of microarray results by RT-qPCR were further used to support the conclusions derived. 

## Materials and Methods

### Material characteristics

The MWCNT used in this study is a MWCNT- XNRI-7; lot 05072001K28, Hadoga Chemical industry (formerly known as Mitsui). In this study it will be referred to as Mitsui7. Chemical composition and length of the same [18] and a different batch [23] have been published previously and results are summarized in Table 1. In brief, the average particle length of Mitsui7, as reported by the manufacturer, is 3-5 μm. One gram of Mitsui7 corresponds to 3.55 x 10^11^ particles and consists of particles with an average width of ca. 100 nm. Transmission Electron Microscopy (TEM) revealed that the median length of Mitsui7 is 3.86 μm with 27.5% of Mitsui7 particles having lengths longer than 5 μm [23]. The numbers of walls vary from 20 to 50 [18]. Major impurities as determined by the collision type Inductively Coupled Plasma Mass Spectrometry, Combustion Ion Chromatography and trace metal analysis included Fe: (0.3%), Na: 0.4% S: ca. 470 ppm and Cl: ca. 20 ppm (18) [23]. 

**Table 1:** Known characteristics of MWCNT-Mitsui-7.

**References**	**Dispersion**	**Tube length**	**Tube Width ± SD**	**Impurities**
Mitsui, Tokyo, Japan	ND	Segments; 3-5 μm long	ND	ND
Tagaki et al. 2008	5% Triton X-100	27.5% longer than 5 μm	100 nm	Fe: 0.35% S: 0.047%
Porter et al. 2010	PBS and dispersion media	Median length: 3.86 μm	49±13.4 nm	Fe: 0.32% Na: 0.41%

ND: Not Determined

### Characterization of Mitsui7 in dispersion medium

#### Scanning Electron Microscopy

Size and the level of agglomeration was evaluated by Scanning Electron Microscopy (SEM) using a Zeiss Ultra 55 (Carl Zeiss Microscopy GmbH, Germany) SEM equipped with a field emission electron source. High vacuum conditions were applied and a secondary electron detector was used for image acquisition. Samples were prepared on a carbon tape surface and coated with a conductive layer of Au with approximately 20 nm thickness. Mitsui7 as dry pristine powders and dispersed in the exposure media, was analyzed by SEM. 

### Preparation of Mitsui7 exposure stock

#### For in vitro exposures

 Immediately before the treatment, Mitsui7 suspensions were prepared as follows. A total of 8.9 mg of Mitsui7 was suspended in 2.6 ml of cell culture media to a total stock concentration of 3.4 mg/ml. Particle suspensions were prepared by sonication of Mitsui7 stock concentrations using a S-450D sonifier (Branson Ultrasonics Corp., Danbury, CT, USA) equipped with a disruptor horn (Model Number: 101-147-037) for a total of 4 minutes at amplitude of 10%, with alternating 10 s pulses and pauses on ice. 

#### For in vivo exposures

Particles were suspended by sonication in 1 ml of MilliQ water containing 0.9% NaCl and 10% v/v acellular broncho alveolar lavage (BAL) fluid collected from C57BL/6 mice. Naïve C57BL/6 mice were flushed twice with 0.6 ml of 0.9% NaCl to obtain approximately 1 ml of BAL. BAL was centrifuged at 400 G for 10 min at 4°C to remove cells and the supernatant was used in the dispersant liquid. The Mitsui7 (4.05 mg/ml) suspension was sonicated on ice using a Branson Sonifier S-450D equipped with a disruptor horn (Model number: 101-147-037) for a total of 4 minutes at amplitude of 10%, with alternating 10 s pulses and pauses. This suspension was used for the high dose (162 µg) and diluted 1:3 in vehicle for the medium (54 µg) dose and diluted further 1:3 for the low dose (18 µg). Between the dilutions the suspensions were mixed by pipetting. Vehicle control solutions were prepared with 0.9% NaCl and 10% acellular BAL fluid and sonicated as described above. 

### Dose selection

Table 2 summarizes the doses chosen for *in vivo* and *in vitro* exposures. 

**Table 2:** Comparison of *in vivo* and *in vitro* doses.

***In vivo* exposure doses**				***In vitro* exposure concentrations**			
µg/animal	162	54	18	µg/ml	100	25	12.5
mg/kg	8.1	2.7	0.9	mg/kg	NR	NR	NR
(assuming an average mouse weighs 20 g)							
mg/kg mouse lung	591	197	65.7	mg/kg	NR	NR	NR
(assuming an average lung weighs 274 mg)							
µg/cm^2^ (lung surface: 82 cm^2^)	1.98	0.66	0.22	µg/cm^2^ (4-wells Petri dish: 21.8cm^2^, 6.8 ml)	31.19	7.8	3.9

NR: Not Relevant

#### *In vivo*

Mice were exposed to 18, 54 or 162 µg of Mitsui7/animal by single intratracheal instillation (these doses are also referred to as low, medium, and high respectively, in the text where appropriate). The doses were selected based on our previous studies [32-36] and reflect 8 h time weighted average (TWA) for carbon black particles (3.5 mg/m^3^) in an occupational setting in Denmark. The lung of an average female C57BL/6 mouse of 20 g weighs 274 mg with an average surface area of 82 cm^2^. Thus, using surface area of exposure as a parameter, the doses chosen in the study can be expressed as 0.22, 0.66 and 1.98 µg of Mitsui7/cm^2^ of lung surface area, respectively. 

#### *In vitro*

Cells were exposed to 12.5, 25.0 and 100 µg Mitsui7/ml (these doses are also referred to as low, medium, and high respectively in the text where appropriate). These concentrations were selected based on the concentration ranges used in the literature that are demonstrated to induce some degree of cellular response, but that are well below the concentrations that induce cytotoxicity (data not shown). Monolayers were exposed in a 4-well Petri dish consisting of 21.8 cm^2^ total surface area. The exposure was carried out in a total volume of 6.8 ml. The *in vitro* concentrations amount to 3.9, 7.8 and 31.19 µg/cm^2^ of Petri dish. Thus, the highest *in vivo* dose (1.98 µg/cm^2^) was approximately half of the lowest *in vitro* concentration used (3.9 µg/cm^2^), based on µg Mitsui7/cm^2^ area. 

### Cell culture, exposure and sample collection

The development of the Muta^TM^Mouse lung epithelial cell line (FE1) and its characterization has been previously described [37]. The FE1 epithelial cells were cultivated in DMEM/F-12 + GlutaMaxTM-1 (Fisher Scientific Biotech Line A/S, 21765037, Slangerup, Denmark) supplemented with 2% fetal bovine serum (In Vitro AS, B1-04-007-1A, Fredensborg, Denmark), 1% penicillin/streptomycin solution (In Vitro AS, B1-03-031-1B, Fredensborg, Denmark) and 100 ng/μl murine epidermal growth factor (Sigma-Aldrich, E4127-1MG, Broendby, Denmark). Cells were maintained in T150 bottle (Thermo Fisher Scientific, NUN-156502, Roskilde, Denmark) at 37.0°C with 5% CO_2_ and 90% humidity. Media was replaced every 2-3 days to ensure proper growth conditions. 

FE1 cells were seeded at 10^5^ cells/ml density to a total of 6.81 ml/well in the 4-well plates (NUNC, Thermo Scientific, Hvidovre, Denmark, 167-063) and incubated at 37°C for 24 hours. The next day cells were examined for overall natural appearance and growth. At the time of treatment, cells showed ~ 80% confluency. Cells were incubated with 0, 12.5, 25, and 100 µg Mitsui7/ml for 24 h. Following the incubation, cells were trypsinized and centrifuged to collect cell pellets. The cell pellets were stored at -80°C until further use. Three individual experiments were conducted (cells were exposed in separate experiments). 

### Animals

#### Ethics Statement

All procedure complied with EC Directive 86/609/EEC and Danish laws regulating experiments on animals (permit 2010/561-1779). Mice did not display any signs of respiratory distress, lethargy or any other physical symptoms following exposure.

Female C57BL/6 mice aged 5-7 weeks were obtained from Taconic (Ry, Denmark). The mice were allowed to acclimatize for 1-3 weeks before the experiment. All mice were given food (Altromin no. 1324, Christian Petersen, Denmark) and water *ad libitum* during the whole experiment. Mice were group-housed in polypropylene cages with sawdust bedding at controlled temperature 21±1°C and humidity 50±10% with a 12-h light:12-h dark cycle. Experiments were initiated at 8 weeks of age. 

#### Animal exposure and tissue collection

Mice were intratracheally instilled as previously described for other nanomaterials [33,38]. In brief, mice were anesthetized by subcutaneous injection of 0.2 ml of Hypnorm® (fentanyl citrate 0.315 mg/ml and fluanisone 10 mg/ml, Janssen Pharma) and Dormicum® (Midazolam 5 mg/mL, Roche) in sterile water. The mice were kept on their back at a 40-degree angle on a hot plate (37 °C) during the entire procedure. Mice were exposed to 18, 54 and 162 µg of Mitsui7 *via* a single intratracheal instillation. A 40 µl suspension was instilled followed by 150 μl air with a 250 µl SGE glass syringe (250F-LT-GT, MicroLab, Aarhus, Denmark). Control animals were instilled with vehicle (0.9% NaCl with 10% BAL). After the instillation catheter was removed, breathing was observed in order to assure that the delivered material did not block the airways. Mice were allowed to recover for 24 hours before sacrifice. For tissue collection, mice were anaesthetized with Hypnorm/Dormicum as described above and killed by exsanguination via intracardiac puncture. The right lung lobes were snap-frozen in cryotubes in liquid N_2_ and stored at -80°C.

All animal procedures followed the guidelines for the care and handling of laboratory animals established by the Danish government. The Animal Experiment Inspectorate under the Ministry of Justice approved the study (#2010/561-1779). 

### Additional animal exposures and tissue collection for validation by RT-qPCR

The doses chosen for both *in vivo* (18, 54 and 162 µg of Mitsui7/animal) and *in vitro* (12.5, 25, and 100 µg of Mitsui7/ml) were high [18], therefore, additional animal exposures to lower doses of Mitsui7 were carried out. Mice were exposed to 2 and 6 µg of Mitsui7 via single intratracheal instillation and samples were collected at 24 hours following the exposure. *In vitro*, cells were incubated with 0.00125, 0.125 and 1.25 µg of Mitsui7/ ml and cells were harvested at 24 hours post-exposure time point. Exposure of animals and sample collection is described above. Tissue samples collected from this exposure were used for validation by RT-qPCR.

### Transmission Electron Microscopy

For transmission electron microscopy (TEM), mice were euthanized and lungs were fixed for a week in a 2% glutaraldehyde buffered fixative (pH 7.2) *via* trachea. Fixed lung was cut in small pieces and a standard Electron Microscope embedding procedure was carried out. Samples were rinsed in 0.15 M phosphate buffer followed by a wash with 0.15 M sodium cacodylate. Post-fixation and osmofication were carried out in 2% osmium tetroxide in 0.05 M potassium ferricyanide for 2 hours. Following osmofication, samples were rinsed in deionized water and placed in 1% uranyl acetate in water overnight at 5 °C. The following day samples were gradually dehydrated in ethanol and lastly in propylene oxide. Embedding was performed in propylene oxide diluted Epon, until 100% Epon 812 was used before polymerisation at 60 °C for 24 hours. Samples were cut into approximately 80 nm sections for TEM using an ultramicrotome with a diamond knife. Sections were stained with uranyl acetate and lead citrate, and imaged using a CM 100 BioTwin instrument from Philips operated at 80 kV accelerating voltage.

### BAL cell counts

Infiltration of inflammatory cells into lung lining fluid was determined by BAL done immediately after cardiac puncture and collection of blood. The statistical analyses were performed in SAS version 9.2 (SAS Institute Inc., Cary, NC, USA). Statistical significance was calculated using a non-parametric one-way ANOVA with a post-hoc Tukey-type experimental comparison test.

### Total RNA extraction

Total RNA was isolated from cell samples (n = 6 replicates per concentration) and from lung tissue of 72 mice in total (n = 6 mice per dose group). Isolations were done using TRIzol reagent (Invitrogen, Carlsbad, CA, USA) and purified using the RNeasy MiniKit (Qiagen, Mississauga, ON, Canada ) as described by the manufacturer. An on-column DNase treatment was applied (Qiagen, Mississauga, ON, Canada). All RNA samples showing A260/280 ratios between 2.0 and 2.15 were further analysed for RNA integrity using an Agilent 2100 Bioanalyzer (Agilent Technologies, Mississauga, ON, Canada). RNA integrity numbers above 7.0 were used in the experiment. If the RNA samples didn’t reach the criteria, new RNA extractions from the lung tissue or cell culture were performed. Total RNA was stored at -80°C until analysis.

### Microarray Hybridization

Total RNA (200 ng) from each sample (n=6 per group) and from universal reference RNA (UMRR, Stratagene, Mississauga, ON, Canada) was used to synthesize double-stranded cDNA and cyanine labeled cRNA using the Agilent Linear Amplification kit (Agilent Technologies, Mississauga, ON, Canada ). Reference RNA was labeled with cyanine 3-CTP and experimental samples were labeled with cyanine 5-CTP (Agilent Technologies Inc., Mississauga, ON, Canada). The cyanine-labeled cRNA was *in vitro* transcribed using T7 polymerase and purified using RNeasy mini kits (Qiagen). Sample and reference targets (825 ng) were combined and hybridized to Agilent 8 x 66K oligonucleotide microarrays (Agilent Technologies Inc., Mississauga, ON, Canada) for 17 hours at 65°C. The arrays were washed according to supplier instructions and then scanned on an Agilent G2505B scanner at 5 µm resolution. Data were acquired using Agilent Feature Extraction software version 9.5.3.1

### Statistical Analysis of Microarray Data

A reference design [39-41] with the sample labelled with Cy5 and the reference labelled with Cy3 was used to analyze the data. The quantified data for each array was read into R [42,43]. The median signal intensity without background subtraction was used in this analysis and probes with technical replicates were averaged using the median. The background for each array was measured using the (-)3xSLv1 probe. Spots with median signal intensities less than the trimmed mean (trim = 5%) plus three trimmed standard deviations of the (-)3xSLv1 probe were flagged as below detection (i.e., absent). The total number of flagged spots, the median signal intensity and standard deviation for the (-)3xSLv1 probe for each array were recorded. Other microarray level summary statistics included the median signal to noise ratio (log_2_ scale) and the median signal intensity for each channel as well as the median absolute deviation. Differentially expressed genes between the control and treated groups were determined using the MAANOVA library [44]. The ANOVA model included treatment and a slide effect. This model was applied to the log_2_ of the relative intensities. The Fs statistic [45], a shrinkage estimator for the gene-specific variance components was used. The p-values for all the statistical tests were estimated using the permutation method (30,000 permutations with residual shuffling). These p-values were then adjusted for multiple comparisons by using the false discovery rate approach (FDR) [46]. Estimated marginal means, also known as least square means [47,48], were estimated for each group. These means are a function of the model parameters and are adjusted for the other factors in the model such as day of hybridization. The least square means were then used to estimate the fold change for each contrast that was tested.

### Quantitative Real Time (qRT)-PCR array validation

Fifty-four individual genes from each cells and tissues were selected for further validation by RT-qPCR using custom RT^2^ Profiler PCR Arrays and a BioRad CFX96 Real-Time PCR detection system. The genes selected were statistically significant in the DNA microarray analysis for at least one condition and were associated with three biological functions: oxidative stress, inflammation and fibrosis. A custom RT^2^ Profiler PCR Array plate, the RT^2^ First Strand Kit and RT^2^ SYBR^®^ Green qPCR Mastermix (QIAGEN Sciences, Maryland, USA) were used. Hypoxanthine-guanine phosphoribosyltransferase (*Hprt*), actin β (*Actb*) and glyceraldehyde 3-phosphate dehydrogenase (*Gapdh*) were used as reference genes for normalization and were selected based on their stable expression levels in the treated and control samples in the microarray results. A threshold value was set to 10^2^. The final RT-qPCR validation group consisted of a sample size of n=3 for both cells (individual passages) and tissues (individual animals).

## Results and Discussion

We exposed mice and mouse lung epithelial cells in culture to three different doses of Mitsui7 to evaluate the efficacy of *in vitro* models to predict *in vivo* outcomes following exposure to ENM. Lung tissues and cell lysates were collected 24 hours post-exposure. Global gene expression profiling was employed to characterize the molecular differences and similarities between *in vivo* and *in vitro* models as they relate to the existing mechanistic information available on the MWCNT-induced toxic effects.

### Dose and concentration selection

*In vivo* doses chosen for this study were based on our previous studies and reflect 1, 3, and 9 days of 8 h time weighted exposure limit for carbon black particles (3.5 mg/m^3^) in an occupational setting in Denmark. *In vitro* doses were chosen to mimic the total lung surface area available for exposure to particles (Table 2). At present, there are no occupational exposure limits specified for MWCNT in Denmark. Taking into consideration the MWCNT aerosol levels found in an occupational environment, the mass median aerodynamic diameter of MWCNT, and the surface area of human alveolar epithelium, Porter et al. [18] calculated that exposure of mice to 10 µg MWCNT is close to one month of human exposure to aerosol concentrations of 400 µg/m^3^ MWCNT in the work environment [18,49]. Mice exposed to 10 µg of MWCNT exhibit transient inflammation, which subsides at 7 days post-exposure. In contrast, mice exposed to 20 and 40 µg MWCNT displayed prolonged inflammation up to 56 days post-exposure, and fibrosis-like conditions are observed following exposure to 80 µg MWCNT [18,50]. The Mitsui7 instillation doses used in the present study span this entire exposure range [18,50].

An aerosol concentration of 440 µg/m^3^ is approximately equivalent to human alveolar epithelium exposed to 226 µg/m^2^ MWCNT per month, which is less than 1 µg/ml concentration for cells exposed in culture medium [18]. Thus, according to these calculations, the *in vitro* Mistui7 concentrations chosen in the present study are approximately an order of magnitude higher than potential human exposures (the lowest concentration being 3.9 µg/ml), as well as approximately an order of magnitude higher than the mouse *in vivo* exposures. However, we note that the lowest *in vitro* dose (12.5 µg) is similar to the highest *in vivo* dose (162 µg) delivered. These very high doses were chosen because they reflect the present literature on MWCNT-induced effects.

### Mitsui7 characteristics in the dispersion medium

The particle size of Mitsui7 in the *in vivo* and *in vitro* exposure dispersion medium was determined using dynamic light scattering (DLS; File S1). The DLS technique measures spherical hydrodynamic diameter and since Mitsui7 exhibit rod-like shapes, it is not clear what the size-spectra of Mitsui7 generated by DLS corresponds to. Size scatter may very well include signals arising from more than one MWCNT dimension and metal impurities; however, the sizes may reflect the hydrodynamic equivalent size distributions. The distribution used in this study is number size distribution. The DLS is used only as an indication of the successful dispersion of the MWCNT and caution must be exercised when interpreting DLS data.

The DLS of unfiltered Mitsui7 in the *in vivo* exposure medium (0.9% NaCl with 10% BAL) showed a slightly skewed unimodal size distribution with a peak size at ca. 5000 nm (Figure S1A). The average zeta size was 8719.8 nm. The polydispersity index (PDI) was 1.0, indicating the presence of large Mitsui7 agglomerates of different sizes. After filtration through a 3.1 μm filter, DLS showed a multimodal size distribution with peak sizes at ca. 600 nm, 1000 nm, and 2000 nm, and an average zeta size of 1701.8 nm, thus indicating the presence of smaller agglomerates (Figure S1A). A PDI of 0.658 was found, demonstrating better dispersion after filtration. The *in vivo* exposure medium was analyzed separately and showed a bimodal size distribution with peak sizes at ca. 85 nm and 250 nm, an average zeta size of 389.9 nm (Figure S1B), and a PDI of 0.717, indicating protein agglomerates of various sizes. Filtration of the exposure medium through a 0.2 μm filter revealed the presence of smaller proteins with peak sizes of 25 nm and 55 nm, and an average zeta size of 67.2 nm. 

The *in vitro* exposure medium (DMEM-F12 cell culture medium with 2% fetal bovine serum) was also analyzed with and without Mitsui7. The analysis of unfiltered Mitsui7 in the *in vitro* exposure medium showed a unimodal size distribution with a peak size at ca. 370 nm (Figure S1C). The average zeta size was 368.6 nm and the PDI was 0.406, indicating well-dispersed Mitsui7. A separate analysis of *in vitro* exposure medium alone showed a unimodal size distribution with a peak size of 5.5 and a zeta average of 14.5 (Figure S1D). 

**Figure 1:**
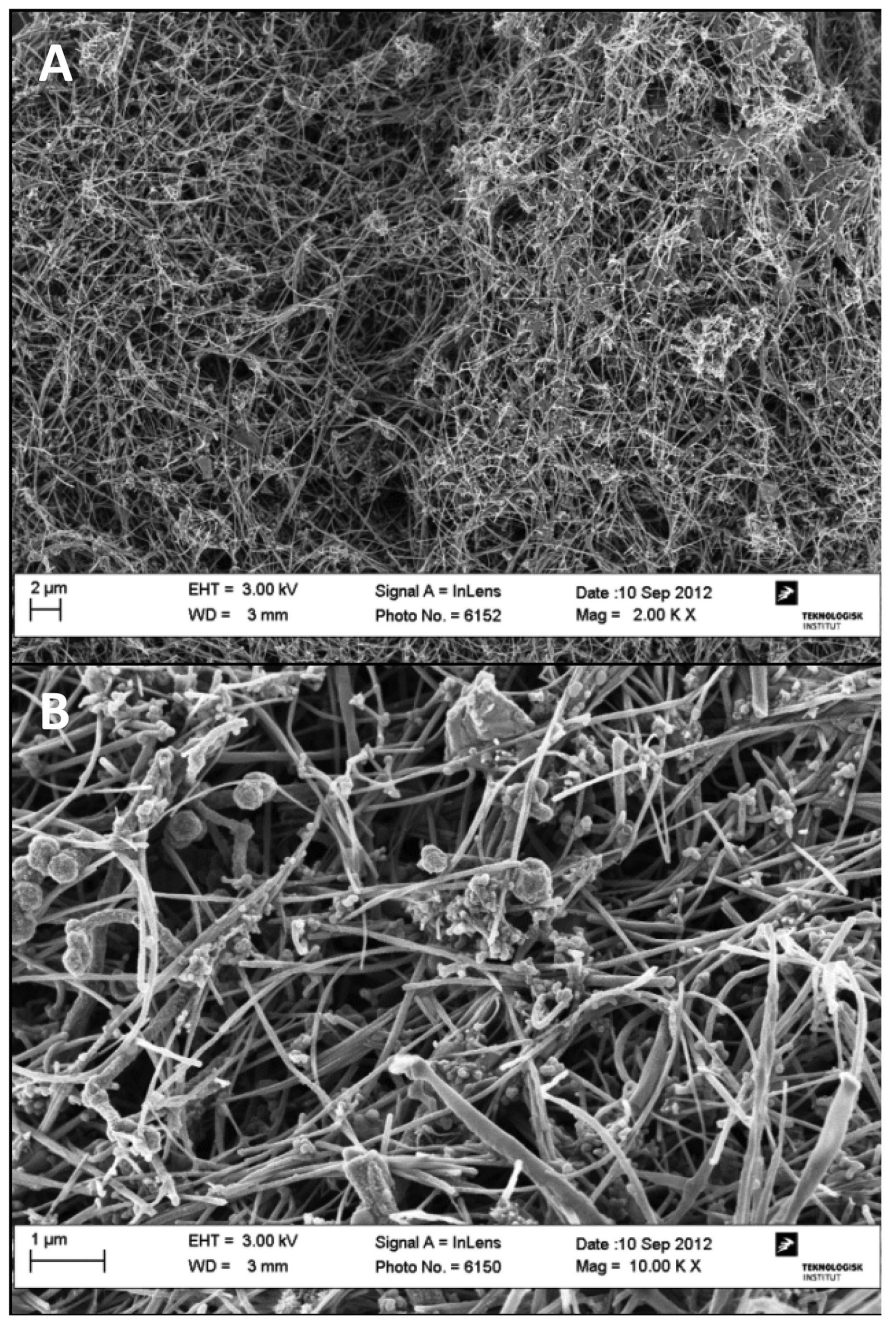
SEM images of Mitsui7 in the *in* *vivo* exposure medium. (A) High density behavior of Mitsui7 (B) Long, straight, and bended tubes are visible. Impurities seen on the MWCNTs may be dried materials from the exposure medium.

**Figure 2:**
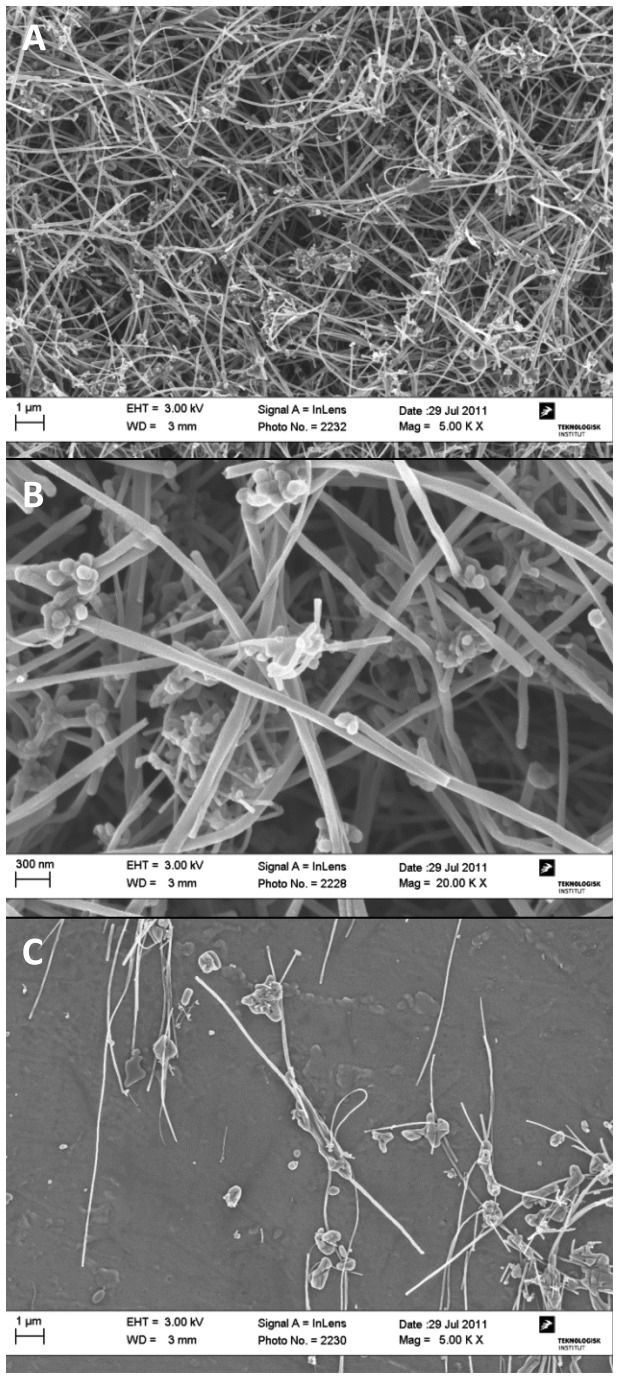
SEM images of dry, pristine Mitsui7. (A) Mitsui7 at high density. (B) A close-up of MWCNT at high density. (C) Mitsui7 behavior at low density.

**Figure 3:**
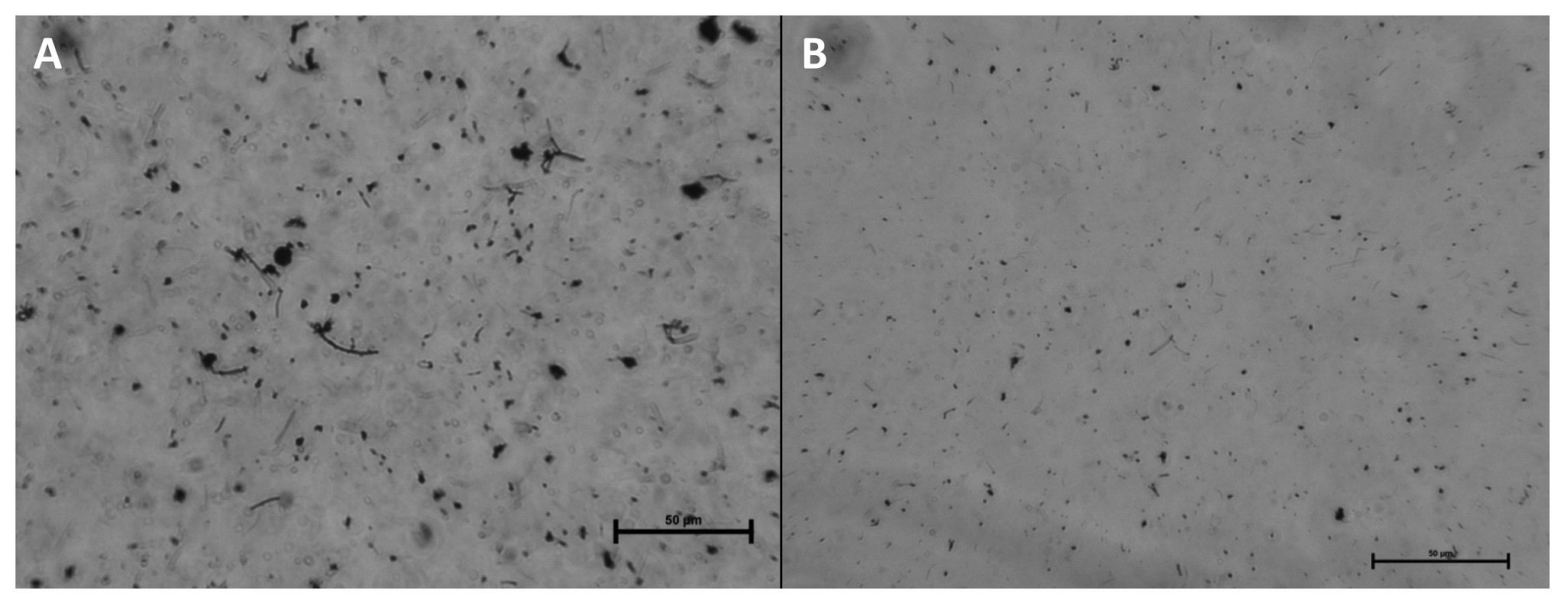
Light microscopy images of Mitsui7 dispersed in the *in* *vitro* exposure medium after sonication. (A) 100 μg/milliliter (31.19 µg/cm^2^) concentration (B) 12.5 μg/ml concentration (3.9 µg/cm^2^). Bars represent 50μm.

SEM was performed on both pristine Mitsui7 and Mitsui7 in the exposure medium used for both *in vivo* and *in vitro* exposures. At all magnifications Mitsui7 appeared intertwined and agglomerated, with both long and straight, and bended tubes (Figures 1A and 1B). Crystalline impurities were observed on the Mitsui7 that may have originated from the dried exposure medium. SEM images of the pristine Mitsui7 are shown in Figure 2A-C. At low quantities, the pristine Mitsui7 appeared long and straight, but at the higher densities agglomerates were observed, with somewhat intertwined and bended tubes. Figures 3A and 3B show light microscopy images of Mitsui7 dispersed by sonication in the *in vitro* exposure medium. Samples appeared well-dispersed at all doses. Cytotoxicity following exposure to the indicated doses of Mitsui7 was assessed *in vitro* previously and revealed no toxicity (NR Jacobsen, unpublished).

**Figure 4:**
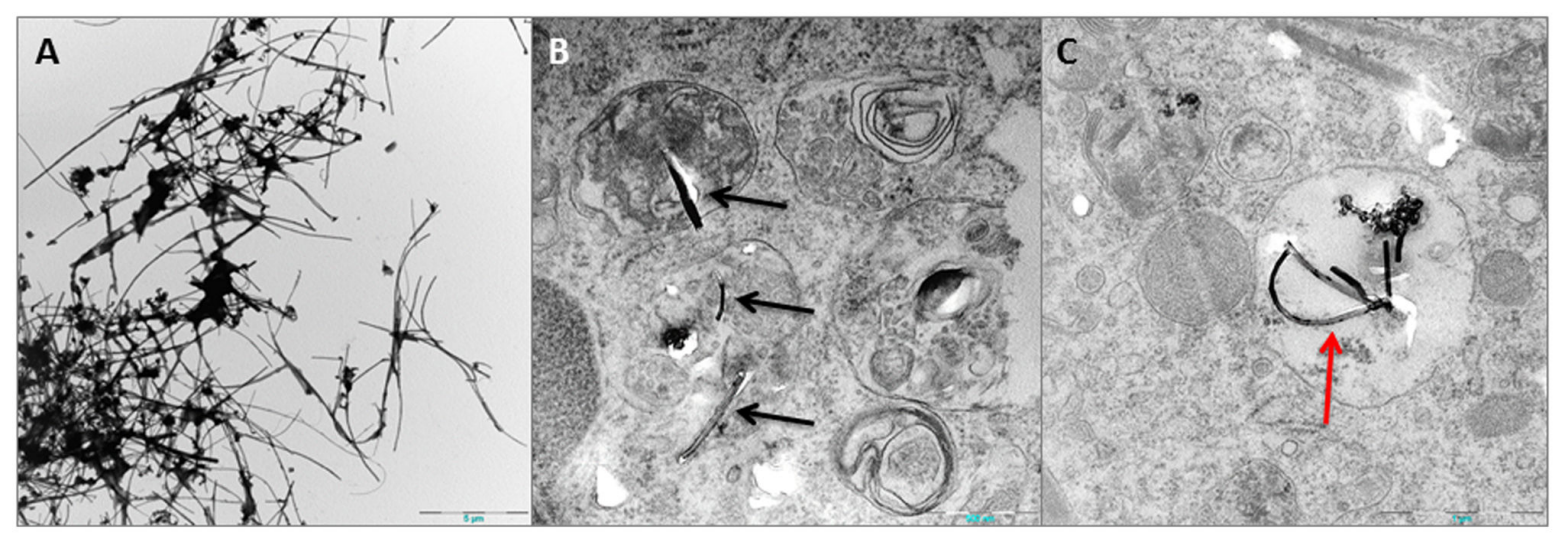
TEM images. (A) Pristine Mitsui7 (B) Mitsui7 MWCNTs interacting with cells in the lung lining fluid (Black arrows). The MWCNTs have been partially dislodged during ultramicrotomy (C) Macrophage containing engulfed Mitsui7 in vesicle (red arrow).

TEM was performed on both pristine Mitsui7 tubes and in the lung lining of the mice intratracheally instilled with Mitsui7. As with SEM, the pristine Mitsui7 appeared long and straight, and although agglomeration was observed, the rigid structure remained (Figure 4A). Diameter of the pristine tubes was measured to 74.4 nm (SD 27.2). Images of Mitsui7 found inside a cell three days after exposure are shown in Figures 4B, the MWCNTs have been partially dislodged during ultramicrotomy. An image of Mitsui7 enclosed in an alveolar macrophage three days after exposure are shown in Figures 4C. No tubes are seen penetrating the macrophage, but several tubes appear to have been engulfed and are enclosed in vesicles. This clearly shows interaction between Mitsui7 and inflammatory cells in the alveolar region. 

### BAL fluid cellular composition

BAL fluid was collected from Mistui7-instilled mice 24 hours after the exposure to assess the recruitment of inflammatory cells into the lung lumen. The total number of cells, neutrophils, macrophages, eosinophils, and lymphocytes were counted and the results are shown in Table 3. The lung inflammatory response was characterized by an increase in the total number of cells that was predominantly the result of a dose-dependent increase in neutrophils (7.2 - 18.2-fold increase over controls). Although a statistically significant increase in the total number of eosinophils was observed in all the dose groups compared to the vehicle treated controls (129.3, 77 and 7.3-fold increase at 18, 54 and 162 μg doses (0.22, 0.66 and 1.98 µg/cm^2^), respectively), an inverted dose response was found, with the highest eosinophil influx found at the lowest dose. Specifically, a 17.6-fold reduction in the number of eosinophils was observed from the lowest dose to the highest dose. A similar trend was observed for the total number of lymphocytes; there was a non-significant increase in the number of lymphocytes in the BAL of the low dose group mice (1.8 fold increase over matched controls) that decreased significantly with increasing dose (0.4 fold compared to matched controls at the high dose). In contrast, the total number of macrophages decreased in a dose-dependent manner relative to controls with a significant decrease found in the highest dose group (0.6 fold) compared to the vehicle treated matched controls. 

**Table 3:** Differential cellular counts in the bronchoalveolar lavage fluid.

**Cell types**	**Dose (µg/animal)**			
	**Control**	**18**	**54**	**162**
Neutrophils x 103 (%)	7.7 ± 1.7 (10.4)	55.1 ± 10.6 (38.6)*	60.6 ± 4.5 (44.5)*	140.2 ± 31.9 (71.8)*
Macrophages x 103 (%)	53.2 ± 2.5 (72.1)	38.2 ± 5.1 (26.8)	37.4 ± 7.4 (27.8)	30.3 ± 8.5 (15.5)*
Eosinophils x 103 (%)	0.3 ± 0.1 (0.4)	38.8 ± 10.7 (27.2)*	23.1 ± 11.1 (17.1)*	2.2 ± 0.5 (1.1)*#
Lymphocytes x 10^3^ (%)	1.6 ± 0.5 (2.2)	2.8 ± 0.5 (2.0)	1.7 ± 0.5 (1.3)	0.6 ± 0.3 (0.3)*#
Total BAL Cell x 103	73.8 ± 3.6	142.8 ± 22.3*	134.7 ± 19.5*	195.3 ± 40.8*

Statistically significant (P<0.05) difference between (*) treated and controls, and (#) low (18 µg) and high (162 µg) doses.

Our results showing increased neutrophil and decreased macrophage populations are consistent with Ma-Hock et al. [11]. However, these studies did not show increases in eosinophils. Although a sharp increase in the total number of eosinophils following exposure to low dose of Mitusi7 may be indicative of allergic airway inflammation [51], it is not clear from the observations why eosinophilia decreased with increasing dose. A similar decline in the total number of eosinophils in the lungs of rats exposed to increasing doses of nano-SiO_2_ was reported [52]. In a murine model of asthma, polymerized liposome nanoparticles were shown to mimic physiological ligand 1 and bind to P selectin on activated airway endothelium. Furthermore, competitive binding of nanoparticles to P selectin was shown to inhibit eosinophil activation resulting in reduced eosinophil-mediated inflammatory response, a hallmark of allergic airway disease [53]. These results support the hypothesis that high doses of Mitsui7 could be competing with physiologic- ligand 1 (required for activation of P selectin) to bind to P selectin on the activated endothelium thereby inhibiting the recruitment of eosinophils into the BAL. A similar argument can also be made for the observed decreases in the lymphocyte population at higher doses.

### Microarray analysis

All microarray data have been deposited in the NCBI Gene Expression Omnibus database and can be accessed through the accession number GSE47000. A total of 4,259 of the 60,000 probes on the array were expressed (where expressed is defined as probes having signal intensities above background in 4 out of 5 samples in at least one experiment condition) in both cells and tissues. Differentially expressed genes were identified using MAANOVA; a probe was considered to be differentially expressed if its normalized signal intensities in treated samples were significantly different from control values following FDR adjustment, with P ≤ 0.05, and the relative change in expression (fold change) was at least ±1.5 in either direction.

### Overview of *in vivo* microarray results

**Figure 5:**
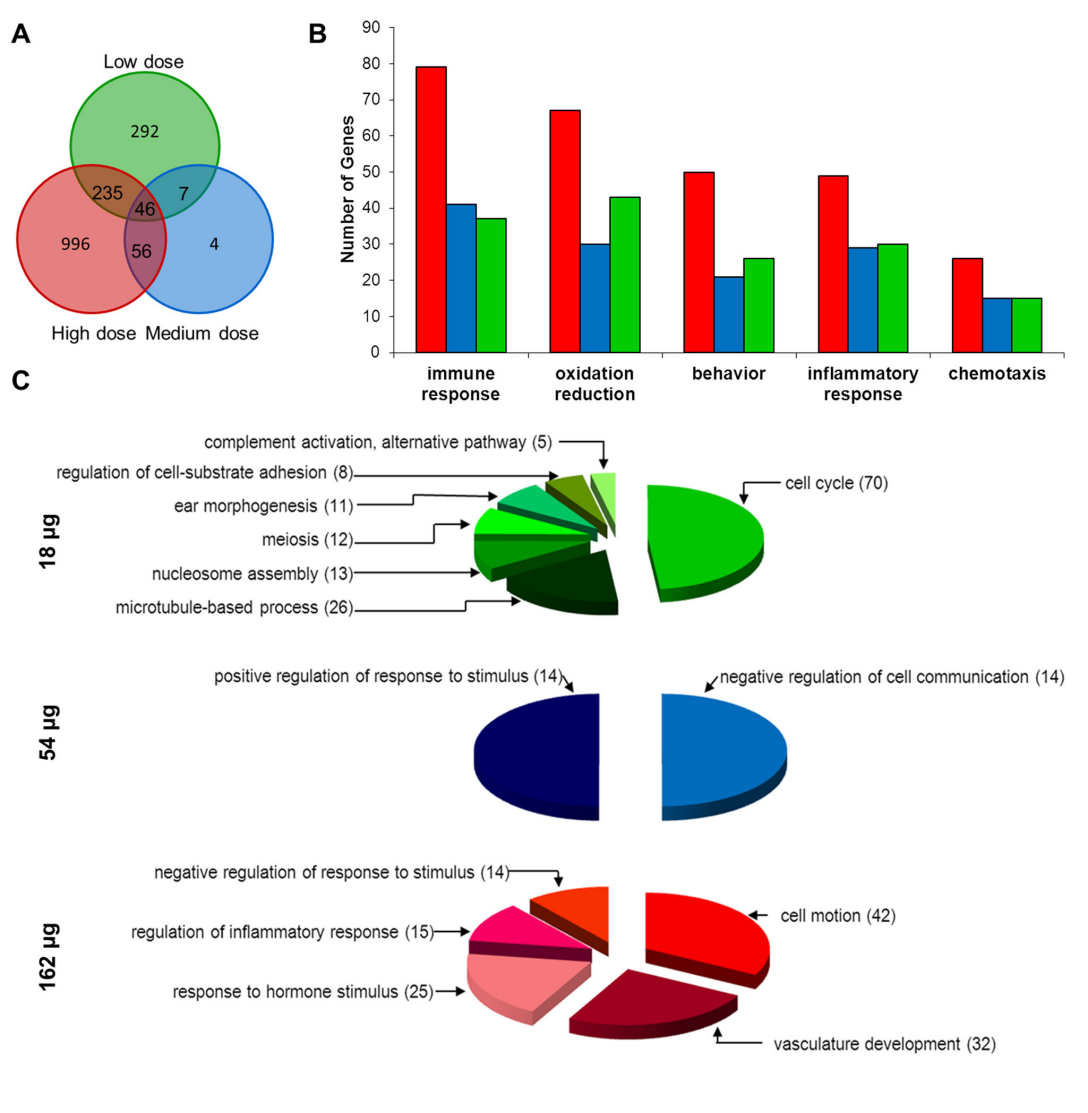
Overview of response in the lung tissue. (A) Venn diagram showing the overlapping differentially expressed genes (FDR P ≤ 0.05 and fold change ≥ 1.5) in response to low (18 µg, green), medium (54 µg, blue), and high (162 µg, red) doses of Mitsui7 in the lung tissues. (B-C) Gene ontology (GO) analysis of differentially expressed genes. (B) Commonly regulated GO biological processes, and (C) GO biological processes unique to the low, medium, and high dose groups. Numbers in parentheses represent number of genes altered in that process.

In general, a high degree of reproducibility was observed within control and treated replicates. A total of 1635 unique genes represented by 2104 probes were significantly differentially expressed in the lungs following exposure to 18, 54, and 162 µg of Mitsui7 at the 24 hours post-exposure time point. A total of 601 (383 genes down-regulated and 219 up-regulated), 114 (35 down-regulated and 79 up-regulated) and 1353 (754 down-regulated and 601 up-regulated) genes were differentially expressed in the 18, 54, and 162 µg dose groups, respectively. These genes are listed in the Table S1. Only 46 genes were commonly differentially expressed in all three dose groups (Figure 5A). Most of the genes in this list exhibited a dose-response and were mainly associated with acute phase response, immune cell trafficking, lung hypersensitivity, and hematological system functions. Increased expression of acute phase response genes (including *Saa1*, *Saa3* and *Timp1*) has also been reported in the lungs of mice exposed to nano-sized titanium dioxide [54,55] and nano- particles of carbon black [56], suggesting acute phase genes are sensitive markers of exposure to nanoparticles [57]. A list of the significant genes that were responsive in all dose groups is provided in Table 4. 

**Table 4:** Differentially expressed genes common across all three doses.

**Gene symbol**	**Entrez ID**	**Average fold change compared to matched controls**		
		**18 µg/mouse**	**54 µg/mouse**	**162 µg/mouse**
*A130040M12Rik*	319269	1.8	2.3	2.7
*Adamts4*	240913	1.8	2.4	3.5
*Adm*	11535	1.9	2.2	3.0
*Asgr1*	11889	-2.2	-3.1	-2.3
*Bcl3*	12051	1.6	1.7	1.7
*Ccdc129*	232016	-3.0	-2.3	-2.7
*Ccl11*	20292	3.0	5.2	3.4
*Ccl17*	20295	3.5	3.4	2.8
*Ccl2*	20296	3.6	5.3	4.0
*Ccl7*	20306	5.5	6.9	4.3
*Ch25h*	12642	2.7	3.1	4.0
*Cldn8*	54420	-2.0	-1.8	-2.0
*Ctps*	51797	2.0	2.7	2.9
*Cxcl1*	14825	2.3	3.9	4.7
*Entpd8*	72090	-2.0	-2.1	-2.4
*Gm2045*	100039096	2.1	2.5	4.4
*Gm2371*	100039687	1.7	2.1	2.8
*Gm3459*	100041663	2.0	2.7	3.9
*Gm4148*	100042984	2.0	2.1	3.0
*Gm4382*	100043353	2.1	1.9	2.5
*Gm4499*	100043524	2.0	2.5	3.6
*Gprasp1*	67298	-1.6	-1.7	-2.6
*Has1*	15116	1.9	2.3	1.8
*Hbegf*	15200	1.8	2.6	2.0
*Il6*	16193	2.6	3.9	6.2
*Klhl24*	75785	-1.9	-1.9	-1.8
*Mt2*	17750	2.7	11.7	27.5
*Myb*	17863	-1.9	-2.1	-2.2
*Osbpl6*	99031	-1.8	-1.6	-1.9
*Rgs16*	19734	1.8	2.1	2.5
*Rrbp1*	81910	1.6	1.6	1.6
*Serpina3g*	20715	3.2	3.4	3.4
*Serpinf1*	20317	-1.7	-1.7	-1.7
*Six4*	20474	-1.7	-1.7	-2.1
*Slc26a4*	23985	9.0	7.7	5.3
*Slc30a2*	230810	-2.2	-1.9	-1.9
*Slc39a14*	213053	1.6	2.3	3.7
*Saa1*	20208	3.8	7.0	12.3
*Saa3*	20210	9.9	12.3	45.6
*Timp1*	21857	7.7	9.7	11.7
*Tnfrsf12a*	27279	1.6	2.4	2.7
*Tnfsf9*	21950	2.0	2.1	2.4
*Trib1*	211770	1.5	1.7	1.6
*Trmt61a*	328162	1.8	1.8	1.7
*Tubb6*	67951	1.8	1.8	1.6
*Tyro3*	22174	-1.6	-1.6	-2.0

We analyzed the gene ontology (GO) classifications of all of the differentially expressed genes to identify themes in the global pulmonary gene expression patterns following exposure to Mitsui7. The processes affected by MWCNT are shown in Figures 5B and 5C, and include ‘common’ (i.e., occurring in more than one group) and ‘unique’ (i.e., occurring in one dose group only) processes, respectively. Processes involved in chemotaxis [GO: 6935], immune response [GO: 6955], inflammatory response [GO: 6954], oxidative reduction [GO: 55114] and behavior [GO: 7610] were over-represented among all the dose groups (Figure 5B). In addition, several unique GO processes were over-represented within a dose group (Figure 5C). In the low dose group, the top biological processes altered consisted of 70 genes involved in cell cycle and was associated with five specific GO terms: cell cycle [GO: 7049], cell cycle process [GO: 22402], cell cycle phase [GO: 22403], M phase [GO: 279], and cell division [GO: 51301]. The other over-represented biological processes in the low dose group included: microtubules [GO: 7017], nucleosome assembly [GO: 6334], meiosis [GO: 7126], ear morphogenesis [GO: 42471], regulation of cell substrate adhesion [GO: 10810], and complement activation, alternate pathway [GO: 6957]. In the medium dose group, negative regulation of cell proliferation [GO: 42127] and positive regulation of response to stimulus [GO: 48584] were uniquely enriched with 14 genes in each of the processes. Unique biological processes affected in the high dose group included: cell motion [GO: 6928], vasculature development [GO: 1944], response to hormone stimulus [GO: 9725], regulation of inflammatory response [GO: 50727], and negative regulation of response to stimulus [GO: 48585]. 

In order to understand the functional significance of these GO changes, Ingenuity Pathway Analysis (IPA Reference) was employed to identify biological networks, functions, and pathways affected in all of the dose groups. Table 5 shows a summary of the top functions and processes altered in the three dose groups 24 hours following the exposure to Mitsui7. This analysis revealed that biological functions such as, cellular movement, cellular growth and proliferation, and cell-to-cell signaling were commonly affected across the dose groups. Closer examination of these differentially expressed genes under each of the functional categories in common revealed that they are actually specifically involved in immune and inflammation related responses. Analysis of biological pathways revealed significant enrichment of genes in the pyrimidine ribonucleotides *de novo* synthesis, aryl hydrocarbon receptor signaling, and LPS/IL-1 mediated inhibition of RXR signaling pathways in the low dose group. In the medium dose group, genes involved in the acute phase response signaling, chemokine signaling and IL-17 signaling pathways were significantly enriched. Finally, retinol biosynthesis, neuregulin signaling, and acute phase response signaling were the most affected pathways in the high dose group. 

**Table 5:** Significantly enriched canonical pathways and networks in *in vivo* exposure.

	**Canonical Pathways**		**Networks**	
**Dose Group**	**Name**	No. of Genes*	**Name**	No. of Genes*
18 μg	Pyrimidine ribonucleotides de novo biosynthesis	7	Organismal injury and abnormalities	27
	Aryl hydrocarbon receptor signaling	13	Cell-to-cell signaling and interaction, immune cell trafficking	25
	LPS/IL-1 mediated inhibition of RXR function	16	Cancer, respiratory disease, cell cycle	22
	Glutathione-mediated detoxification	4	Embryonic development, organ development, organismal development	22
	LXR/RXR activation	9	Cell cycle, cancer	19
	Estrogen-mediated S-phase entry	4	Cellular assembly and organization, cellular function and maintenance, connective tissue development and function	18
54 μg	Acute phase response signaling	9	Cellular movement, immune cell trafficking	24
	Chemokine signaling	5	Cancer, organ morphology	16
	IL-17 signaling	5	Organismal injury and abnormalities, inflammatory response	16
	Pyrimidine ribonucleotide de novo biosynthesis	3	Lipid metabolism, molecular transport, small molecule biochemistry	6
	LXR/RXR activation	5	Cell morphology, cell-mediated immune response	1
	Retinoate biosynthesis I	3	Cell-to-cell signaling and interaction, cellular assembly and organization	1
162 μg	Retinol biosynthesis	10	Cellular movement, cellular assembly and organization, cellular function and maintenance	33
	Neuregulin signaling	16	Cellular movement, cell death and survival, drug metabolism	30
	Acute phase response signaling	23	Infectious disease, organismal injury and abnormalities, cell morphology	29
	Role of tissue factor in cancer	16	Lipid metabolism, molecular transport, small molecule biochemistry	29
	Aldosterone signaling in epithelial cells	20	Cellular development, cellular growth and proliferation	28
	Glucocorticoid receptor signaling	29	Cell-mediated immune response, cellular development, cellular function and maintenance	28

* Number of genes significantly regulated in each pathway or network

**Figure 6:**
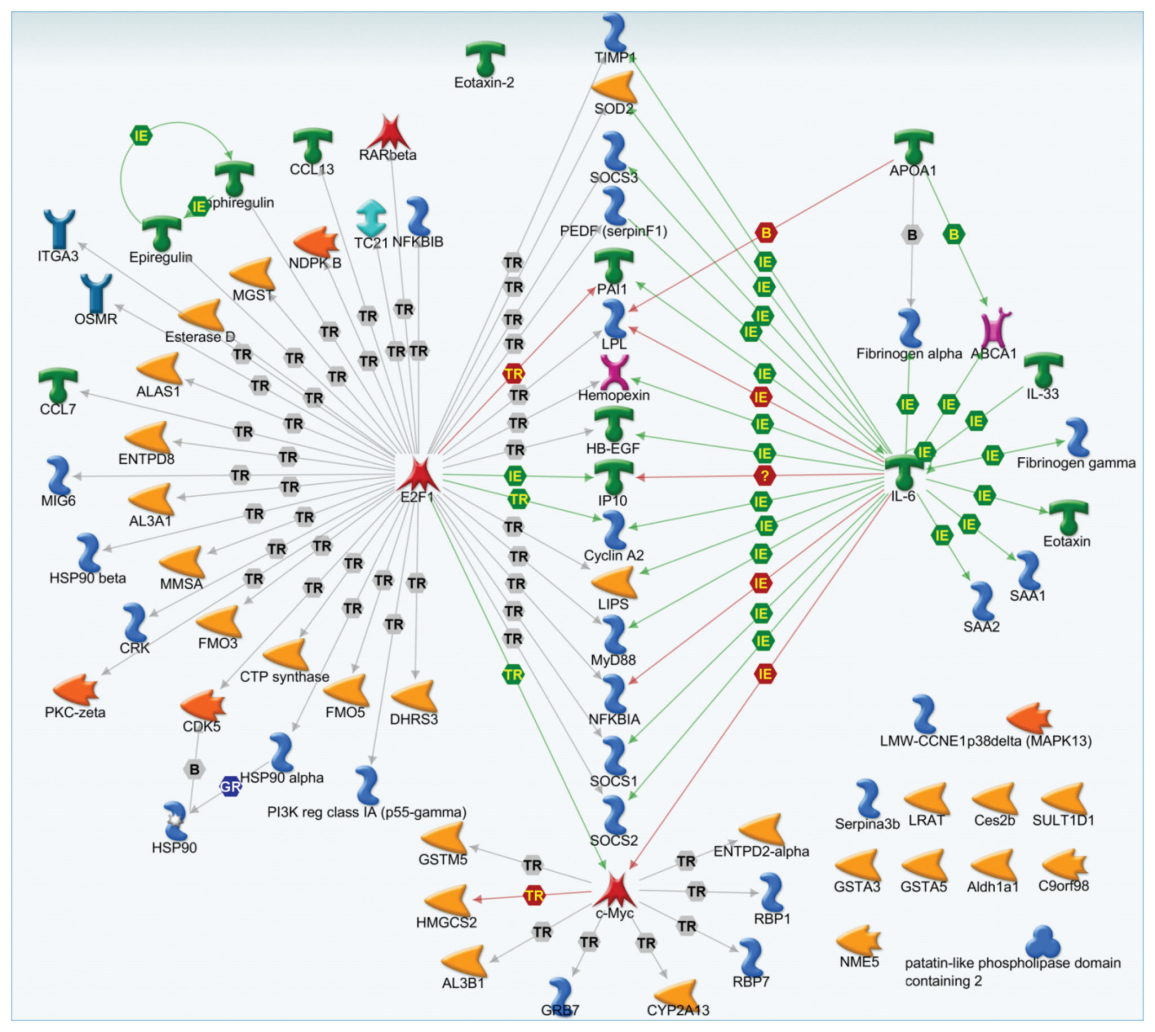
Gene network. Combined analysis of all significantly differentially expressed genes associated with angiogenesis, lipid metabolism, and cellular proliferation pathways in lungs in the high dose group. Analysis was conducted and the network was built using GeneGo Metacore map creator. The figure shows that the majority of the genes are commonly regulated by *E2f1*, *Il6* and *C-myc*. Hexagons represent physical and functional interactions: TR – transcription regulation, IE - influence on expression, B – binding, and GR – group. The green arrows represent positive regulation/activation, the brown arrows represent negative regulation/inhibition and the grey arrows represent unspecified regulation.

A detailed study of a subset of genes implicated in the processes mentioned above revealed dose-specific responses ranging from imbalances in nucleotide synthesis in the low dose to inflammation, angiogenesis and lipid metabolism related effects in the high dose. For instance, we found significant down-regulation of adenylate kinase 8 (-2.1 fold), ectonucleoside triphosphate diphosphohydrolase 2 and 8 (-1.9 and -2.0 fold), and nucleoside diphosphate kinase 5 (-2.1) in the low dose. These genes are actively involved in the synthesis and metabolism of uridine-5’-triphosphate (UTP) and adenosine triphosphate (ATP) nucleotides required for the maintenance of ion homeostasis in the lungs [58,59]. Decreased synthesis and depletion of pyrimidine nucleotides prevents blockade of the epithelial sodium channels, increase alveolar fluid clearance and clearance of lung edema in mice infected with respiratory syncytial virus and influenza A virus [58-60]. Metabolism of adenine nucleotides in human airway epithelial surfaces plays a role in the regulation of mucociliary clearance related epithelial functions. Imbalances in this pathway can lead to inhibition of sodium ion transport leading to accumulation of alveolar fluid and pulmonary edema. Excess ATP in the lung fluids is suggested to contribute to the onset of inflammation in humans suffering from pulmonary pathology including alveolar damage, inflammation and edema (reviewed in 61. Enrichment of *de novo* pyrimidine synthesis pathway in the low dose group may thus reflect a non-specific cellular response to invasion of lungs with particles, possibly an effort leading to their clearance. In the medium dose, a dramatic up-regulation of several acute phase and inflammation associated genes such as *Saa3* (12.3-fold), *Saa1* (7.0 fold), *Saa2* (12.0 fold), *Il6* (4-fold), *Ccl7* (7.0 fold), *Ccl11* (5-fold), *Ccl13* (6.0 fold), and *Ccl24* (10.0 fold) was noted. These changes suggest an active recruitment of inflammatory cells to the site of exposure aiding in clearance efforts. These genes are also affected by the high dose. However, in addition to a significant up-regulation of genes implicated in acute phase and inflammation pathway in the high dose, down regulation of carboxylesterases 2C and 2E (-1.7, -2.1 fold), dehydrogenase reductase (-1.6 fold), retinol binding proteins 1 and 7 (-1.7, -2.4 fold), lecithin retinol acyltransferase (-3.0 fold) and up-regulation of esterase D (1.65 fold), lipoprotein lipase (2.5 fold), lipase-hormone sensitive (2.5 fold), amphiregulin (3.0 fold), epiregulin (2.8 fold) were noted. These genes are involved in functions such as angiogenesis, lipid metabolism, and cellular proliferation. Combined analysis of all genes altered under these pathways using the GeneGo Metacore map creator tool revealed that of the 80 genes associated with the pathways described above, 60 were commonly regulated by *E2f1* (a cell cycle regulatory transcription factor), *Il6* (an inflammatory modulator) and *C-myc* (an oncogene). We note that these regulators were also significantly differentially expressed in the treated groups (Figure 6). 

In general, the above results reveal a clear distinction in tissue response across the doses. The response in the lowest (18 μg or 0.22 µg/cm^2^) dose was marked by a negative regulation of the cell cycle process, in parallel with changes in the nucleotide metabolism and synthesis pathways. Cell cycle arrest and imbalance of nucleotide metabolism can be viewed as early regulatory signals initiating an organism’s defense functions including phagocytic mopping and airway clearance mechanisms in lungs. This was evident in the medium dose (54 μg or 0.66 µg/cm^2^), which showed positive regulation of acute phase response and inflammatory mediators. In the high dose (162 μg or 1.98 µg/cm^2^), the overall response was much greater than the other two dose groups and included simultaneous enrichment of several biological processes including inflammation, acute phase response, angiogenesis, pro-and anti-fibrotic factors, and anti-oxidants. 

### Overview of *in vitro* microarray results

**Figure 7:**
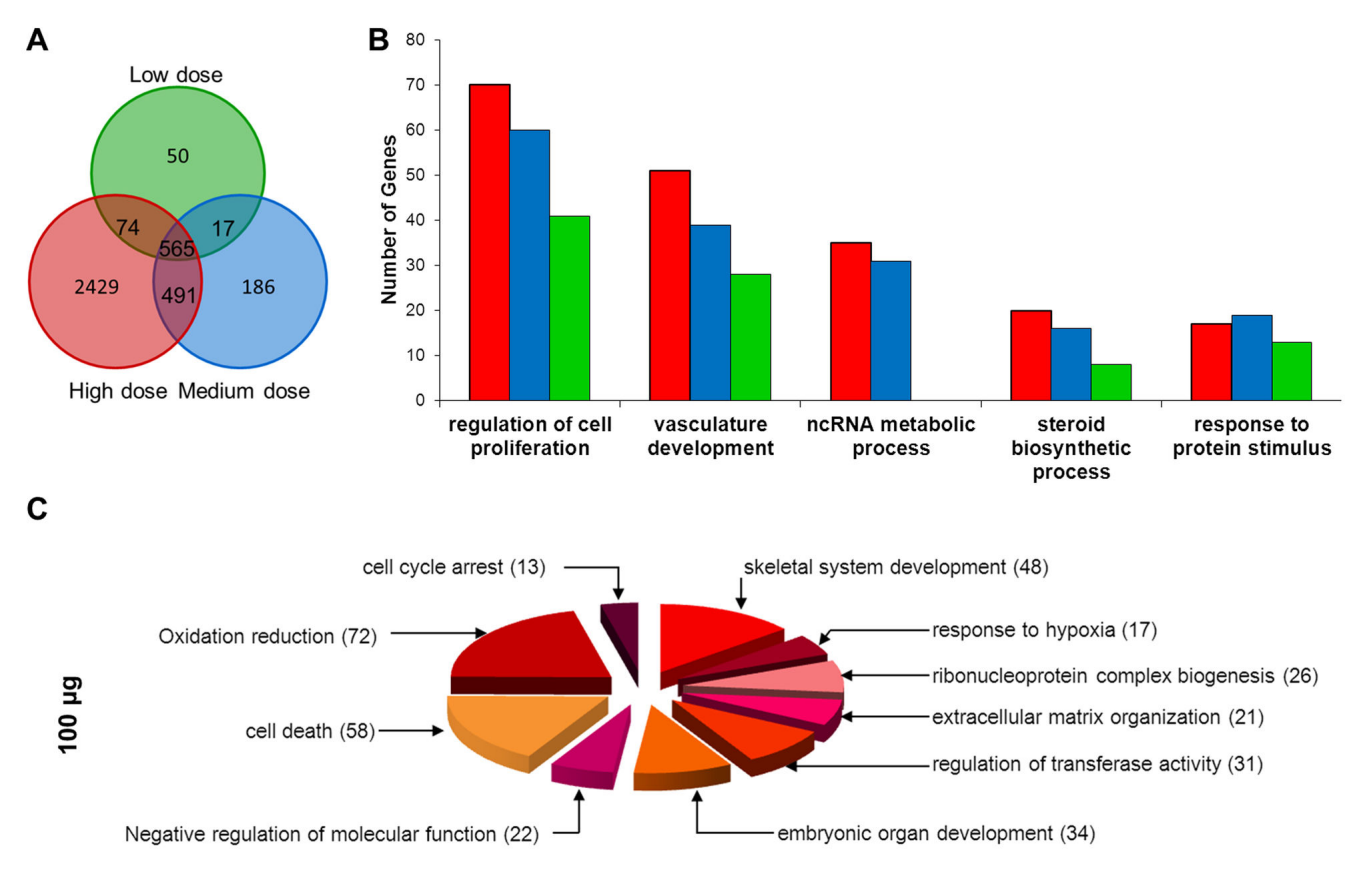
Overview of response in FE1 cells. (A) Venn diagram showing the overlapping differentially expressed genes (FDR P ≤ 0.05 and fold change ≥ 1.5) in response to low (12.5 µg, green), medium (25 µg, blue), and high (100 µg, red) doses of Mitsui7 *in* *vitro*. (B-C) Gene ontology (GO) analysis of differentially expressed genes. (B) Commonly regulated GO biological processes (C) GO biological processes unique to the high dose group. Numbers in parentheses represent number of genes altered in that process.

Lung epithelial cell monolayers were exposed to 12.5 (low), 25.0 (medium) and 100 μg (high) Mitsui7/ml (corresponding to 3.9, 7.8 and 31.19 µg/cm^2^, respectively) for 24h as described in methods and materials. MAANOVA analysis was applied to identify differentially expressed genes. This gene list was filtered to remove probes that were below our detection threshold (i.e., probes that were not expressed above background levels). This analysis revealed a total of 1931 unique genes represented by 2413 probes that were significantly differentially expressed (FDR adjusted P<0.05 and ±1.5 fold change) in the cells exposed to 12.5, 25 or 100 μg Mitsui7/ml (3.9, 7.8 and 31.19 µg/cm^2^). A dose-dependent increase in transcriptional effects was found, with a total of 782 (432 genes down-regulated and 350 up-regulated), 1337 (682 down-regulated and 655 up-regulated), and 1721 (921 down-regulated and 801 up-regulated) genes differentially expressed in the 12.5, 25 and 100 μg Mitsui7/ml (3.9, 7.8 and 31.19 µg/cm^2^) groups, respectively. The full list of significant genes is available in Table S2. A total of 565 genes were affected by every concentration of Mitsui7 (Figure 7A). Most of the genes that were affected in the medium and high concentration groups were also affected in the low concentration group (Figure 7A) and were mainly associated with Aryl hydrocarbon receptor (AHR) signaling, glutathione mediated detoxification, acute phase response signaling, cholesterol biosynthesis, hepatic fibrosis/hepatic stellate activation, and NRF2 mediated oxidative stress response. A list of genes affected in common for every concentration analyzed is provided in Table S3. 

To be consistent with the analysis conducted on *in vivo* microarray results, we employed GO functional classification to assign specific functional or biological processes to the differentially expressed genes following *in vitro* exposure to Mitsui7. The common and unique biological processes significantly enriched in different exposure groups across the dose groups are shown in Figures 7B and 7C. Processes involved in steroid biosynthetic process [GO: 16126], vasculature development [GO: 1944], regulation of cell proliferation [GO: 42127], response to protein stimulus [GO: 51789], and ncRNA metabolic process [GO: 34660] were the commonly over-represented GO groups (i.e., consistently appearing within two or three exposure groups) and consisted of 96 genes. In the medium and high concentration groups these processes were further represented with GO terms such as blood vessel morphogenesis [GO: 48514], angiogenesis [GO: 1525], and positive regulation of cellular proliferation [GO: 8284]. Several GO processes were uniquely altered in the high concentration group and included oxidation reduction [GO: 55114], response to hypoxia [GO: 1666, GO: 70482], cell cycle arrest [GO: 7050], cell death [GO: 8219, GO: 16265, GO: 12501], organ development [GO: 48568, GO: 60348, GO: 42724, GO: 51216, GO: 1501, GO: 48705], extracellular matrix organization [GO: 30198], regulation of activity [GO: 51338, GO: 45859, GO: 43549, GO: 44092], and lipid biosynthetic process [GO: 8610]. Of particular interest, and in contrast to our *in vivo* findings, analysis of GO processes and functions revealed a high degree of overlap across the exposure groups, with exceptions generally only occurring at the highest concentration. This suggests that although the number of genes and the magnitude of change (i.e., fold change) exhibited a dose-response, there was no accompanying concentration-dependent increment in the nature of toxicity. Thus, the results indicate that the lowest concentration itself was capable of inducing a similar response/toxicity as what was observed in the medium and high exposure groups. 

**Figure 8:**
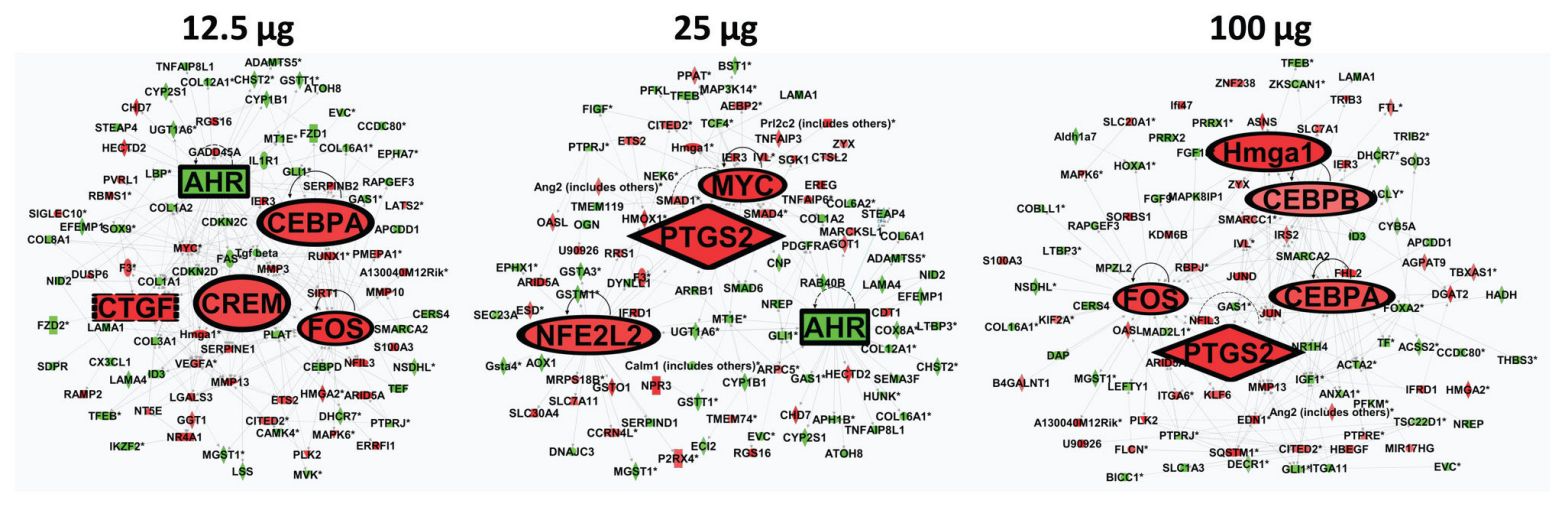
Top three biological networks *in* *vitro*. The networks were generated by merging the top three networks in each of the dose and were based on the connectivity between each gene and the molecular interaction knowledge base in IPA. Nodes are highlighted in large bold font. Green indicates down-regulation and red indicates up-regulation.

To identify potential biological pathways affected by MWCNT exposure in lung epithelial cells, the significant gene list (Table S2) was overlaid onto molecular canonical pathways in IPA. Four major canonical pathways were identified as commonly affected in all the three dose groups: hepatic fibrosis/hepatic stellate cell activation, aryl hydrocarbon receptor signaling, super pathway of cholesterol biosynthesis, and glutathione-mediated detoxification. The majority of the genes implicated in these pathways were down-regulated. Table 6 lists all of the genes involved in these four pathways. Gene interaction networks were then created to visualize the linkages between the different genes and pathways using the IPA network tool. Figure 8 shows the three top scoring networks within each concentration merged into a single concentration-specific meta-network. In the low concentration, organ development, cardiovascular system development and function, and cancer networks were merged. The network consisted of several nodes including: *C-*fos, *Crem1*, *Ctgf*, *Cebpa*, and *Ahr*. In the medium exposure group, cellular growth and proliferation, organismal development and drug metabolism, and glutathione depletion networks were merged. This network consisted of core nodes including; *alpha catenin*, *Ptgs2*, *Ahr*, *Myc*, and *Nrf2*. In the high concentration, cellular development, lipid metabolism, cell cycle and tissue development networks were merged with *Cebpa* and *Cebpb*, *Hmga1*, *Ptgs2*, and *Fos* as the main nodes. 

**Table 6:** Top four canonical pathways affected in all three doses *in* *vitro*.

**Canonical Pathways**	Gene names*
Hepatic Fibrosis/Hepatic Stellate Cell Activation	*Acta2, Ccl5, Col1a1, Col1a2, Col3a1, Ctgf, Cxcr3, Edn1, Fas, Fgf1, Figf, Igf1, Il1r1, Il1rl1, Il4r, Lama1, Lbp, Met, Mmp13, Myh10, Myl6b, Pdgfa, Pdgfc, Pdgfra, Pdgfrb, Rela, Serpinb2, Smad4, Tgfb2, Tgfb3, Tnfrsf11b, Tnfrsf1a, Vcam1, Vegfa*
Aryl Hydrocarbon Receptor Signaling	*Ahr, Aldh1a1, Aldh3b1, Aldh4a1, Aldh6a1, Aldh7a1, Aldh9a1, Ccnd1, Ccnd3, Cdkn1A, Cyp1b1, Esr1, Fas, Fos, Gsta3, Gsta5, Gstm1, Gstm3, Gsto1, Gsto2, Gstt1, Hsp90b1, Hspb2, Jun, Mdm2, Mgst1, Myc, Nfe2l2, Nqo2, Rela, Tgfb2, Tgfb3, Tgm2*
Superpathway of Cholesterol Biosynthesis	*Acat2/Acat3, Dhcr7, Dhcr24, Ebp, Fdft1, Fdps, Hadhb, Hmgcs1, Hsd17b7, Idi1, Lss, Msmo1, Mvd, Mvk, Nsdhl, Pmvk, Sc5dl, Sqle, Tm7sf2*
Glutathione-Mediated Detoxification	*Gsta3, Gsta4, Gsta5, Gstm1, Gstm3, Gsto1, Gsto2, Gstt1, Gstt3, Gstz1, Mgst1*

* Genes associated with each of the pathways are presented.

Overall, *in vitro* lung epithelial cells showed a robust response to Mitsui7 with alterations in numerous critical functions including gene expression (expression of many transcription factors were altered), metabolism, cellular growth and proliferation, and these changes were found in genes involved in numerous potential diseases such as cancer and fibrosis. However, the response was not dose-dependent; major biological processes and functions altered in the high (100 μg/ml or 31.19 µg/cm^2^) concentration were also altered significantly in the lowest (12.5 μg/ml or 3.9 µg/cm^2^) exposure group, suggesting that the lowest dose chosen was sufficiently high to cause the severe cellular damage.

### Comparison of gene expression profiles of *in vivo* lung tissue and *in vitro* lung epithelial cells

**Figure 9:**
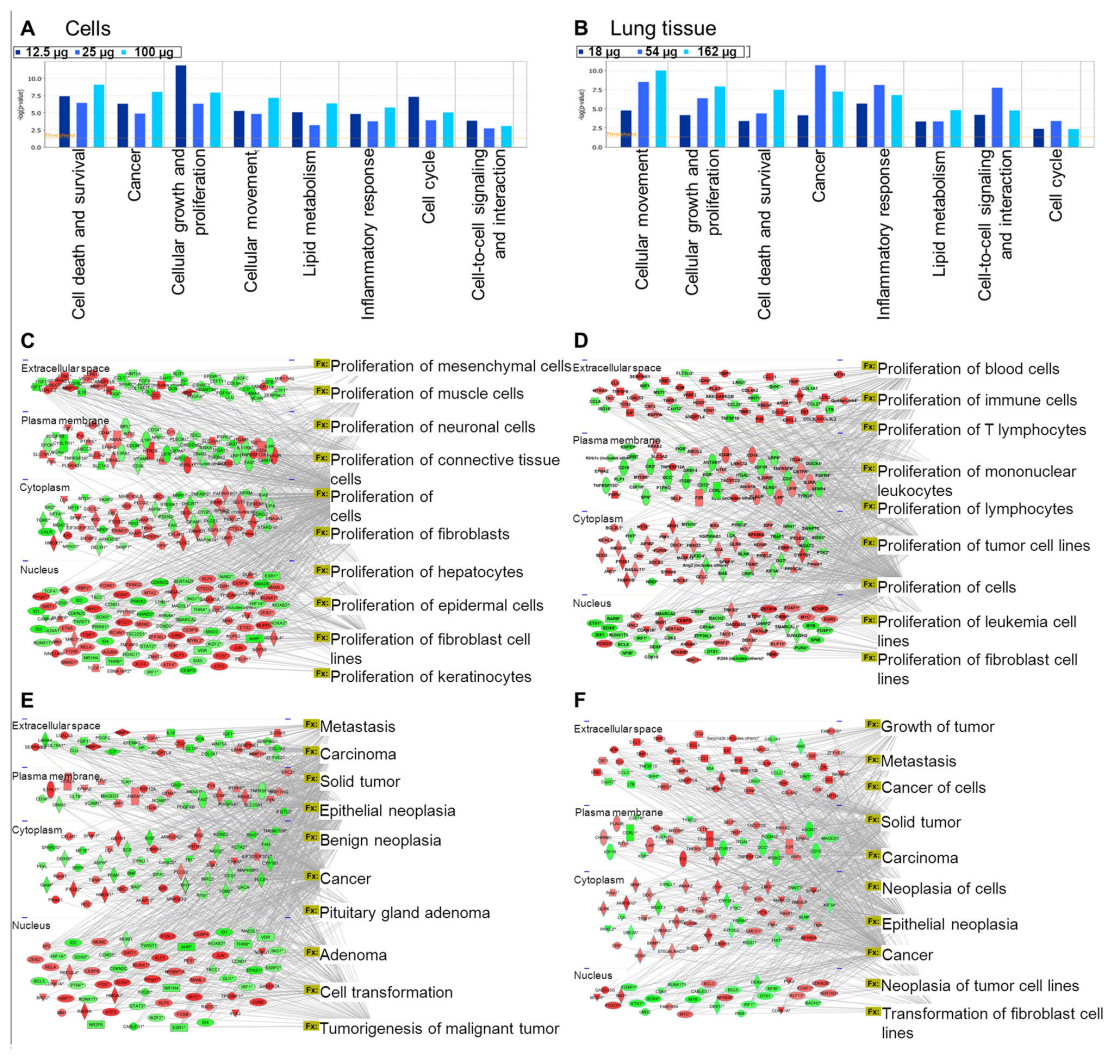
Comparison of biological functions and networks altered *in* *vitro* and *in* *vivo*. Top 10 biological functions altered in lung epithelial cells (A, C, E) and in the lung tissue (B, D, F). The spatial visualization of gene network associated with the biological functions cellular growth and proliferation (C-D) and cancer (E-F). Grey lines connect the significantly differentially expressed genes to the annotation terms associated with each biological function.

In order to determine the relevance of responses observed in cultured lung epithelial cells following exposure to Mitsui7 to *in vivo* lung responses, we analysed all differentially expressed genes (compared to unexposed control) from both cell cultures and tissues using IPA’s function analysis tool. Three categories were included in the analysis: Diseases and Disorders, Molecular and Cellular Functions and Physiological System Development and Function. More than 10 individual high-level processes were significantly enriched and in common to both cell cultures and tissues under each of the three categories. We filtered these high level individual functions by 1) removing redundant functions with overlapping genes, and 2) removing functions that were not directly relevant to the present study (e.g. renal diseases, auditory diseases etc.). The final list of the top most significantly affected high-level biological functions are shown in Figures 9A and 9B. 

**Figure 10:**
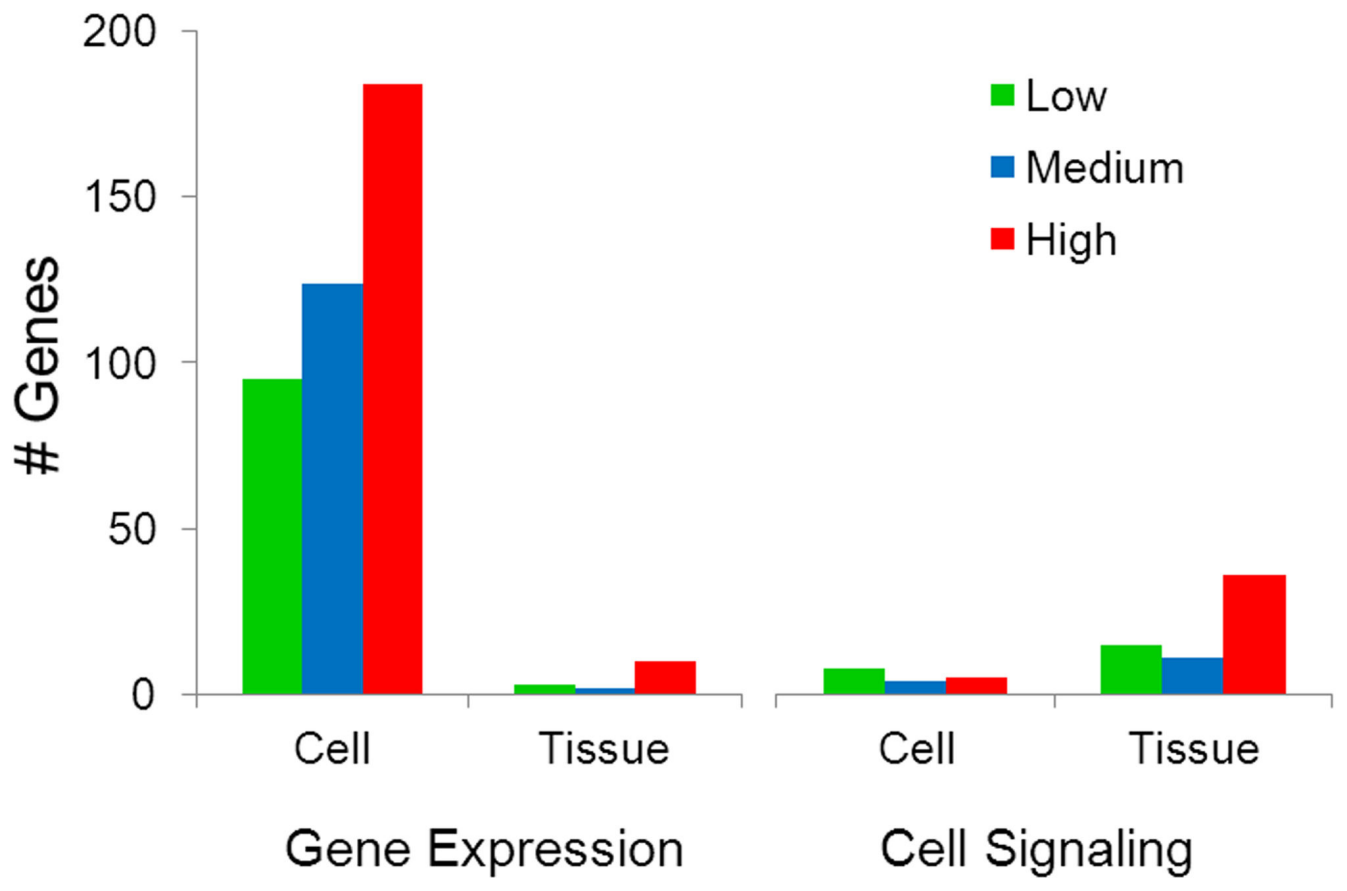
The total number of genes perturbed in the categories of gene expression and cellular signalling functions. Green: low dose (12.5 µg *in* *vitro*, 18 µg *in* *vivo*), blue: medium dose (25 µg *in* *vitro*, 54 µg *in* *vivo*), and red: high dose (100 µg *in* *vitro*, 162 µg *in* *vivo*).

Since the objective was to directly compare the responses in both cells in culture and tissues, we chose to analyze two high-level functions in detail systematically. Cellular growth and proliferation was one of the top high-level functions under the Molecular and Cellular functions category. A total of 155, 214 and 274 genes in the low, medium, and high concentrations *in vitro*, and 32, 36 and 211 genes in the low, medium, and high doses *in vivo* were associated with cellular growth and proliferation. In cell cultures, this function was enriched with annotation terms such as proliferation of cells, muscle cells, fibroblasts, connective tissue cells, and mesenchymal cells (Figure 9C). In the lung tissues however, cellular growth and proliferation function was associated with annotation terms such as proliferation of cells, tumor cells, immune cells, blood cells and proliferation of mononuclear leukocytes (Figure 9D). We then analysed the high-level function given the name ‘cancer’ that was significantly enriched in both cell cultures and tissues under the Diseases and Disorders category. A total of 103, 163, and 220 genes in the low, medium, and high concentrations *in vitro*, and 60, 40, and 159 genes in the low, medium, and high concentrations *in vivo* were associated with this function. This function was associated with terms such as cancer, epithelial neoplasia, cell transformation, solid tumor, and metastasis both *in vivo* and *in vitro* (Figures 9E and 9F). In tissues, annotation terms included growth of tumour, neoplasia of cells, carcinoma, metastasis and other related terms for this function. Further systematic analysis of all of the genes associated with cellular growth and proliferation and cancer revealed that *in vitro* these genes primarily include transcription factors and transcription regulators. In contrast, genes affected *in vivo* were grouped primarily into two categories involved with growth factors and inflammatory modulators in addition to other secretory molecules. In keeping with this result we found significant enrichment of the function ‘gene expression’ in cells in culture, with > 200 genes in this category compared to approximately 10 genes associated with ‘gene expression’ function in tissues (Figure 10). Our analysis clearly demonstrates that significantly more genes are associated with cell signalling *in vivo* compared to *in vitro* following Mitsui7 exposure. These results indicate that although most of the genes altered in both *in vivo* and *in vitro* belong to the same functional categories, suggesting a similar biological response to Mitsui7, the underlying mechanisms and the outcome of such alteration could potentially be different in the two systems studied.

**Figure 11:**
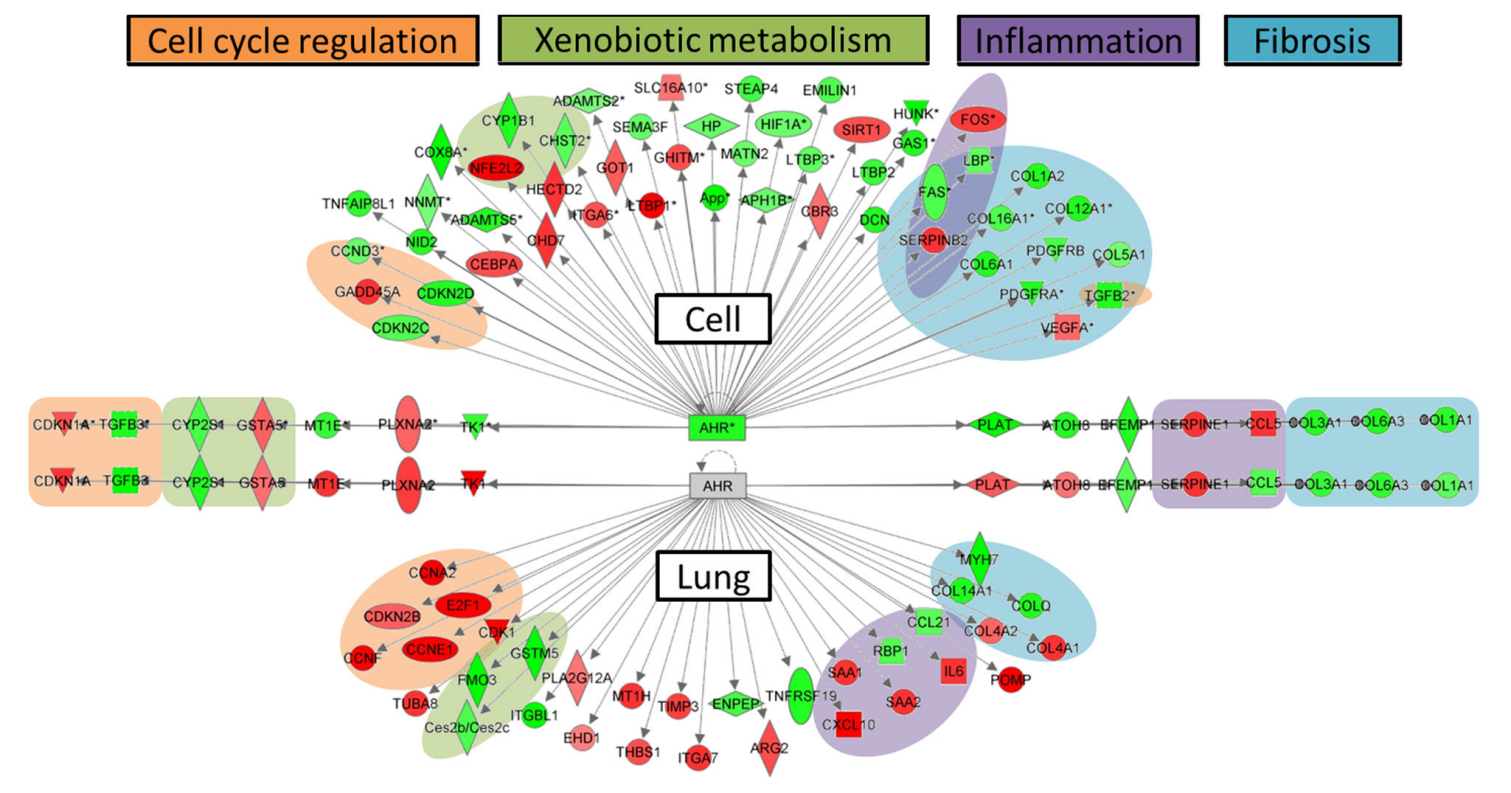
Differentially expressed genes associated with five top canonical pathways derived from IPA analysis. **Genes** **are** **categorized** **into** **four** **major** **functions** (cell cycle regulation-orange, xenobiotic metabolism-green, inflammation-violet, and tissue fibrosis-blue). All genes in both models are regulated by a common transcription factor, AHR. Genes highlighted with green indicate down-regulation and genes highlighted with red indicate up-regulation. The transcription factor AHR is down-regulated in cells, suggesting negative regulation of the downstream functions in cells.

We explored the *in vitro* and *in vivo* data sets to identify potential commonalities in the two systems that may contribute to the same biological outcome, and thus point to the specific relevance of this cell culture model. We conducted a detailed analysis of the responsive genes in the top five significantly enriched canonical pathways affected in both cells and lung tissues. This downstream analysis was limited to the top five affected pathways to minimize the number of interpretations. The top five pathways included: hepatic fibrosis/hepatic stellate activation, AHR signalling, glutathione mediated detoxification, superpathways of cholesterol synthesis, and LPS/IL-1 mediated inhibition of RAR function. Since hepatic fibrosis/hepatic stellate activation and LPS/IL-1 mediated inhibition of RAR function showed a high amount of overlap (i.e., genes functioning in both pathways), we excluded the latter from the analysis. Our examination of all of the genes grouped under each of these pathways revealed that these genes are all regulated by a common nuclear receptor, AHR, and are associated with four major biological processes regulated by the AHR: cell cycle regulation, xenobiotic metabolism, inflammation, and tissue fibrosis (Figure 11). While the common genes involved in cell cycle regulation were similarly up-regulated in both tissues and cell cultures exposed to Mitsui7, genes involved in glutathione mediated detoxification and fibrosis were down-regulated in both systems. Although changes in expression of genes associated with similar functions (cell cycle, inflammation and fibrosis) were observed across all of the concentrations, perturbation of cell cycle and xenobiotic metabolism processes were only observed in the low dose group in the lung tissues, and perturbations in inflammation and fibrosis were only observed at the high dose *in vivo*. These results confirm our earlier interpretation that there was a dose dependent transition in response/toxicity in tissues, but there was no such dose-related transition of biological functions (by IPA) in cell cultures.

**Figure 12:**
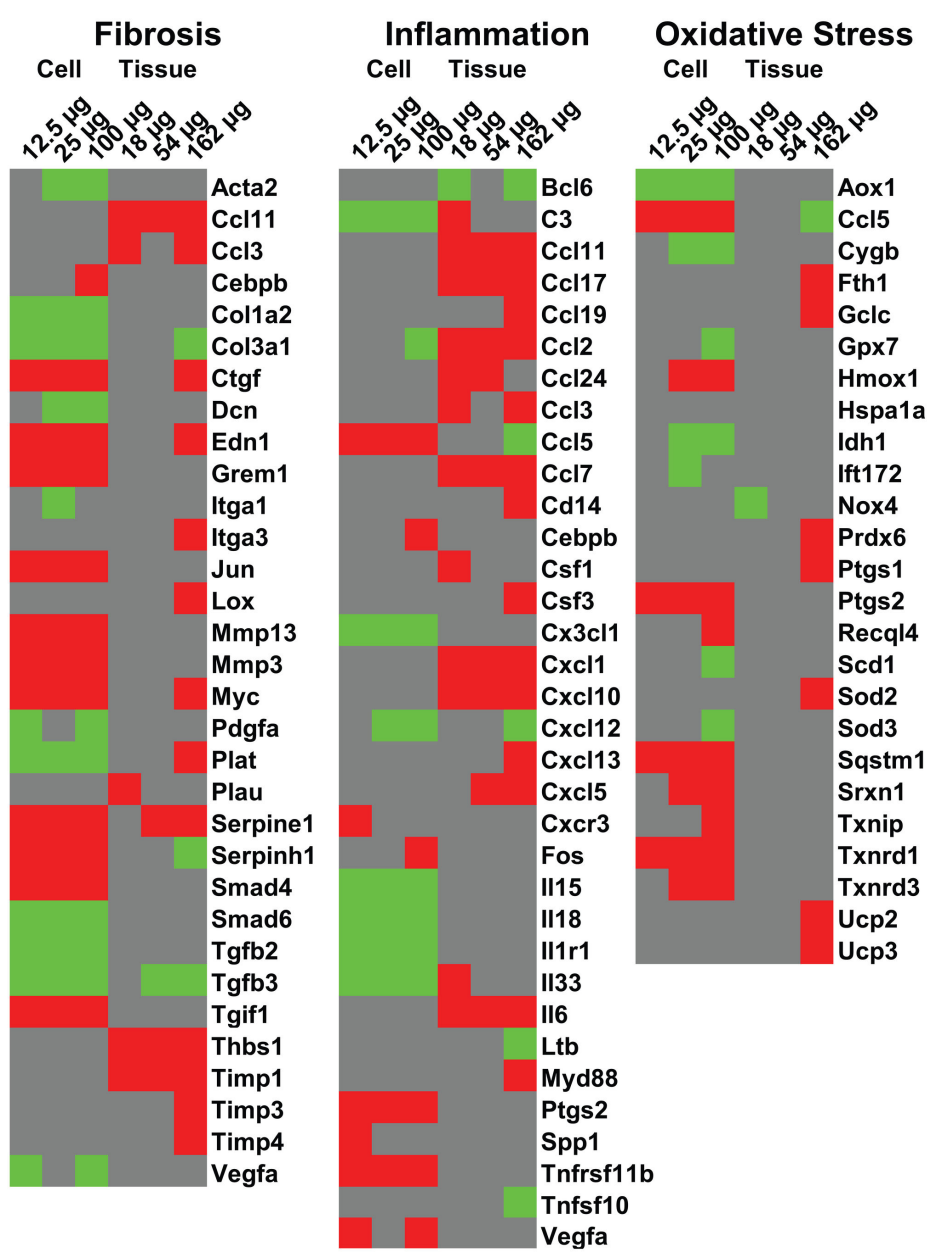
Gene heatmap comparing differentially expressed genes in the categories of fibrosis, inflammation and oxidative stress. Red represents up-regulation, green represents down-regulation and grey represents no change in the expression.

In addition to the analysis described above, we also examined our gene lists for markers of inflammation, oxidative stress, and tissue damage (fibrosis) since these effects are the well-characterized endpoints for both tissues and cells in culture following exposure to MWCNT. More than 100 genes that are known to be associated with each of these characteristic markers of MWCNT were used to build a heat-map (Figure 12) to visualize the similarities and differences between the test systems. Only the genes that showed significant expression changes in either cell cultures and/or the lung tissues treated with Mitsui7 (in at least one exposure group) are shown in color (red up-regulated; green down-regulated; grey no change). Although the total number of genes altered in each of these pathways was somewhat similar between the two models, differentially expressed genes *in vitro* were associated mainly with positive regulation of oxidative stress and fibrosis. In the lung tissues, perturbed genes were associated predominantly with pro-inflammation. Although the lung tissue showed significant alteration in the expression of a number of genes in the fibrosis category, expression profile was indicative of negative regulation of fibrosis. ROS generation and oxidative stress have been proposed to be the primary mode of action of many ENM [1], where a balance between enzyme-mediated ROS generation and ROS detoxification by antioxidant enzymes and the level of intracellular antioxidants such as glutathione regulate intracellular ROS-mediated stress. Up-regulation of antioxidant enzymes including SOD, catalase, GPx and thioredoxin is considered as indication of adaptation to oxidative stress [62]. In the lung tissue, oxidative stress-associated genes, such as *Sod2*, *Ucp2*, and *Ucp3*, were up-regulated. SOD2 is localized to mitochondria and transforms superoxide radicals into hydrogen peroxide that in turn are converted to water and oxygen by catalase or glutathione peroxidase. Uncoupling protein 2 and 3 (UCP-2, -3) are the major components of the electron transport chain. *Ucp* genes act to uncouple the proton gradient in the inner mitochondrial membrane, allowing protons to re-enter the mitochondrial matrix thereby regulating build-up of ROS in cells [63,64]. Similar mitochondrial respiration leakage and up-regulation of mitochondria-specific antioxidants such as SOD2 has been reported in mice intratracheally instilled with SWCNT [65]. The results suggest that the oxidative stress observed *in vivo* was potentially due to mitochondrial respiration leakage. In contrast, *Sod1* and *Sod3* were down-regulated *in vitro* and *Hmox1*, *Sqstm1*, *Srxn1*, *Txnip*, *Txnrd1*, *Txnrd3* were up-regulated. These differences suggest that broadly different mechanisms are driving the oxidative stress *in vitro*. However, it is important to note that the observed differences could also be due to the different time points investigated in the two systems. Further work considering early time points is warranted to rectify these conclusions.

A similar contradiction in the underlying mechanisms involved in the function ‘fibrosis’ was also observed in the two systems. Matrix metalloproteinases (MMPs) were up-regulated in cells in culture, whereas, the expression of inhibitors of metalloproteinases (TIMP) was increased in the lung tissue. A profound increase in the expression of several acute phase stress genes (*Saa*, *Cxcls* and *Ccls*), chemokines, and cytokines was observed in the lung tissue, mainly reflecting chemotaxis and infiltration of inflammatory cells into the lungs. However, in contrast, increased expression of inflammatory genes was associated with epithelial activation (e.g., *Ccl5, Ptgs2*) *in vitro*. Overall, these results suggest different mechanisms, varying degrees of severity in response and potentially different biological outcomes to Mitsui7 exposure in epithelial cells in culture and lung tissue. 

In order to understand if the observed discrepancies between the models were due to basal level differences in the expression of genes in the two systems studied, we compared gene expression profiles of vehicle treated controls of lung epithelial cells and mouse lung tissues. We found that 60% of the genes had comparable expression levels (15,972 of 26,409 analyzed), which included genes implicated in the inflammatory and acute phase responses. Thus, differences in expression were observed for 40% of the genes (Table S4) and these genes were mainly associated with biological processes such as Cellular growth and proliferation, Cancer, Cell death and survival, Cell cycle and Cellular movement in lung epithelial cells, which mainly reflect the consequences of cellular immortalization processes. These results imply that altered basal expression levels in thousands of genes involved in Growth and Proliferation, Cell cycle and other important biological functions may play a critical role in how cells in culture respond to external stimuli such as nanomaterials. The high basal levels of expression of several growth and survival factors may provide protection against cellular injury caused by exposure to nanomaterials. This hypothesis was supported by the observed lack of response in cells treated with very low concentrations of MWCNT (described below, Table 7 and 8). In contrast, changes in the expression of important acute phase and inflammatory genes were observed in lung tissues even at the very low doses. Our results suggest that data from single cell models must be interpreted with caution.

**Table 7:** RT-qPCR validation of genes in lung tissue.

**Genes**	**2μg**	**6 μg**	**18 μg**		**54 μg**		**162 μg**	
	**PCR array**	**PCR array**	**PCR array**	**Microarray**	**PCR array**	**Microarray**	**PCR array**	**Microarray**
*Saa3*	5.5	-	-	9.9	215.8	12.3	348.7	45.6
*Cxcl5*	-	-	66.9	-	188.8	5.3	81.5	4.1
*Cxcl1*	-	-	8.0	2.3	20.5	3.9	22.1	4.7
*Ccl2*	-	3.6	14.5	3.5	-	5.0	21.9	3.9
*Ccl7*	-	8.0	-	5.5	65.9	6.9	20.2	4.3
*Il6*	-	2.0	-	2.8	-	4.0	16.1	6.5
*Timp1*	-	3.0	-	7.7	-	9.7	13.1	11.7
*Ccl11*	-	4.4	-	3.0	15.6	5.2	5.3	3.4
*Saa1*	-	-	-	3.8	-	7.0	5.0	12.3
*Ccl17*	3.1	5.2	7.8	3.5	7.5	3.4	4.9	2.8
*Ccl3*	1.9	-	3.7	1.9	-	-	4.6	2.4
*Lox*	-	-	2.1	-	1.8	-	3.8	2.4
*Myc*	1.5	-	1.9	-	2.0	-	3.0	1.9
*Cd14*	-	-	2.4	-	2.7	-	2.9	2.0
*Ccl19*	-	-	-	-	-	-	2.7	2.5
*Il33*	-	-	-	2.8	3.8	-	2.6	-
*Cxcl10*	-	2.4	-	4.5	13.8	6.0	2.5	-
*Thbs1*	-	-	1.9	-	-	2.9	2.4	2.1
*Ctgf*	-	-	-	-	-	-	2.4	2.0
*Gclc*	-	1.3	2.1	-	-	-	2.2	1.8
*Fth1*	-	-	-	-	-	-	2.0	2.3
*Ptgs1*	-	-	-	-	-	-	1.9	2.8
*Nox4*	-	-	-	-1.8	-3.6	-	-1.7	-
*Ltb*	-	-1.5	-	-	-	-	-2.2	-2.1
*Serpinh1*	-	-	1.8	-	-	-	-2.6	-2.2
*Tnfsf10*	-	-	-	-	-2.3	-	-4.5	-2.8
*Timp3*	-	-1.5	-1.7	-	-2.0	-	-	2.3
*Sod2*	-	-	-	-	-	-	-	1.8
*Ucp2*	-	-	-1.6	-	-2.0	-	-	2.2
*Cxcl13*	-	-	-	-	5.3	-	-	3.8
*Prdx6*	-	-1.8	-2.4	-1.5	-3.0	-	-	1.5
*Ucp3*	-	-1.7	-2.5	-	-2.0	-	-	2.2
*Edn1*	-	-1.7	-	-	-2.5	-	-	1.9
*Itga3*	-	-	-	-	-1.7	-	-	2.1
*C3*	-	1.8	-	2.3	2.8	-	-	-

Only significantly differentially expressed genes are shown (FDR P<0.05, fold change >1.5 in either direction). Data were normalized using *Hprt*, *Gapdh*, and *Actb* as reference genes.

**Table 8:** RT-qPCR validation of genes in cells.

**Genes**	**0.00125**	**0.125**	**1.25**	**12.5**		**25.0**		**100.0**	
	**PCR array**	**PCR array**	**PCR array**	**PCR array**	**Microarray**	**PCR array**	**Microarray**	**PCR array**	**Microarray**
*Mmp13*	-	-	1.6	15.0	9.7	18.2	12.7	27.6	10.6
*Serpine1*	-	-	-	7.3	2.9	5.9	3.9	10.1	5.0
*Ccl5*	-	-	-	2.3	3.0	4.3	2.7	8.0	3.4
*Ctgf*	-	-	-	3.9	3.0	5.4	4.3	7.7	4.6
*Mmp3*	-	-	2.9	33.1	3.5	5.3	3.9	7.0	3.0
*Sqstm1*	-	-	-	4.0	1.8	4.1	2.0	6.2	2.6
*Grem1*	-	-	1.7	2.4	2.5	3.4	2.4	5.5	2.5
*Tgif1*	-	-	-	5.5	2.0	4.4	2.3	5.0	2.5
*Hmox1*	-	-	-	2.7	-	3.1	2.1	4.7	2.5
*Myc*	-	-	-	4.1	1.8	3.0	2.8	4.6	2.6
*Tnfrsf11b*	-	-	-	-1.7	2.1	3.0	2.2	4.3	2.0
*Jun*	-	-	-	-	2.3	2.8	2.8	3.7	3.0
*Ptgs2*	-	-	-	5.4	2.5	2.6	2.9	3.7	2.6
*Spp1*	-	-	-	3.3	2.0	3.2	-	3.5	-
*Fos*	-	-	-1.5	-7.8	-	2.7	-	3.1	1.8
*Vegfa*	-	-	-	-	-	2.2	-	2.7	1.6
*Edn1*	-	-	-	1.5	2.2	2.6	2.5	2.7	2.2
*Txnip*	-	-	-	2.5	-	2.0	-	2.6	1.7
*Txnrd1*	-	-	-	3.1	1.7	2.2	1.9	2.5	2.1
*Srxn1*	-	-	-	-	-	1.7	1.5	2.1	1.5
*Txnrd3*	-	-	-	2.9	-	2.0	1.7	2.1	1.8
*Cebpb*	-	-	-	-	-	-	-	1.8	1.6
*Cygb*	-	-	-	1.7	-	1.6	-1.6	1.8	-1.6
*Cx3cl1*	-	-	-	-	-2.4	-1.8	-2.4	-1.5	-2.3
*Plat*	-	-	-	-	-1.6	-1.8	-1.6	-1.5	-1.5
*C3*	-	-	-	-2.8	-2.0	-1.7	-2.1	-1.7	-2.2
*Il1r1*	-	-	-	-2.2	-1.7	-2.1	-1.7	-1.9	-2.0
*Cxcl12*	-	-	-	-5.6	-	-1.9	-2.3	-2.1	-3.0
*Col3a1*	-	-	-	-8.6	-1.8	-2.1	-2.4	-2.3	-3.3
*Il18*	-	-	-	-9.6	-1.8	-2.5	-2.0	-2.4	-2.4
*Smad6*	-	-	-	-3.6	-1.8	-2.4	-2.1	-2.5	-2.7
*Tgfb3*	-	-	-	-5.0	-	-2.4	-1.9	-2.5	-2.7
*Il15*	-	-	-	-3.0	-1.5	-2.6	-1.5	-2.7	-1.5
*Idh1*	-	-	-	-4.9	-	-2.3	-1.9	-3.0	-2.0
*Dcn*	-	-	-	-4.9	-	-2.7	-3.4	-3.1	-4.7
*Aox1*	-	-	-	-4.5	-2.4	-4.1	-3.2	-3.7	-3.2
*Tgfb2*	-	-	-	-7.9	-1.7	-3.5	-2.3	-4.6	-3.4
*Il33*	-	-	-	-67.3	-3.6	-	-4.4	-13.0	-6.8
*Col1a2*	-	-	-	-5.6	-1.7	-	-2.4	-	-2.8
*Gpx7*	-	-	-	-2.3	-	-	-	-	-1.8
*Pdgfa*	-	-	-	-1.7	-1.5	-	-	-	-1.5
*Cxcr3*	-	-	-	1.6	1.5	1.9	-	-	-

Only significantly differentially expressed genes are shown (FDR P<0.05, fold change >1.5 in either direction). Data were normalized using *Gapdh* and *Actb* as reference genes.

### Validation of microarray results by RT-qPCR

We validated 42 genes altered in cell cultures and 42 genes in tissues (84 in total) categorised under three main established markers of MWCNT-induced effects: oxidative stress, inflammation, and fibrosis. A complete list of genes validated by RT-qPCR is provided in Table S5. Because the exposures used in the DNA microarray analysis are considered to be high compared to the expected exposure levels in the environment, we included a range of lower (lower than 12.5 μg/ml in *in vitro* and lower than 18 μg/mouse *in vivo*) exposure levels for RT-qPCR validation. The final validation by RT-qPCR included 0.0125, 0.125, 1.25, 12.5, 25 and 100 µg/ml in FE1 cells, and 2, 6, 18, 54, 162 µg/mouse *in vivo*. The RT-qPCR results were broadly consistent with the microarray results (Tables 7 and 8). Interestingly, in cell cultures, no significant changes were observed, with the exception of moderate down-regulation of *Mmp3* and up-regulation of *Cx3cl1* at the lowest concentration (0.0125 µg/ml) tested, (we note that the direction of change for *Cx3cl1* was reversed at the higher concentrations). In order to confirm that lack of response in FE1 cells following low concentration exposures was not due to the analysis of only a subset of genes associated with a few biological effects, global analysis of gene expression analysis was performed (as described above) on the lower concentration (0.0125, 0.125, 1.25 µg/ml) groups. The microarray analysis (data not shown) confirmed the subtle response observed by RT-qPCR. In contrast, in the lung tissues, several genes (*Saa3, C3, Cxcl10, Myc, Ccl3, Ccl17, Ccl11, Timp1, IL6, Ccl7, Ccl2 and Cxcl5*) involved in acute phase and inflammation were significantly up-regulated even at the lowest concentrations tested (2 µg/mouse). The data suggest that the lung tissue is generally more sensitive to Mitsui7 exposure than the FE1 lung epithelial cells and that these genes can potentially be used as sensitive markers of exposure to Mitsui7 and like particles. 

### Perspectives

Despite the urgent need for rapid *in vitro* assays that are predictive of *in vivo* toxicity for ENM testing, few *in vitro* validation exercises have been conducted to comprehensively characterize mechanistic similarities and differences for ENM-exposed cells in culture compared to tissues from live model organisms exposed to ENM. Existing publications on this topic have been limited to a small selection of biological endpoints that include inflammation, oxidative stress, and tissue damage (reviewed in 9,66-68). In these existing studies, the *in vivo* lung toxicity assays applied have generally consisted of analysis of BAL fluid for markers of inflammation (infiltration of inflammatory cells, increased production of cytokines and chemokines), oxidative stress (oxidative stress markers, DNA damage), and cytotoxicity (LDH and alkaline phosphatase). These measurements are then analyzed in parallel in cells exposed *in vitro* to the same substance. For example, Wang et al. [15], measured markers of fibrosis in lung tissues and in different lung cell culture models and found good agreement between the profibrogenic responses *in vitro* and *in vivo* [15]. In another study, Horie et al. [69] measured levels of hydroperoxy octadecadienoate (tHODE), an indicator of oxidative stress, in rat lungs exposed to nickel nanoparticles and in cells exposed in Petri culture dishes. They showed comparable increases in tHODE 24 hours after the exposure to particles *in vivo* and *in vitro* [69]. However, although similar perturbations were described *in vitro* and *in vivo* in the above studies, the magnitude of response between the lung tissue and cells in culture in the above studies was different. These differences in the magnitude of response may occur because of difficulties in matching the predicted particle-exposed lung surface area to the surface area of Petri dishes in which the cells are incubated. Moreover, *in vivo* studies evaluated toxicity in complex multi-cellular tissues; whereas, *in vitro* studies primarily focus on understanding the response of a single cell type isolated from a specific organ.

In the present study, we exposed mice *via* instillation and incubated mouse lung epithelial cells in culture dishes to three different doses of Mitsui7. Our *in vivo* to *in vitro* dose comparison was based on the total lung surface area and the total surface area of the Petri dish in which cells were exposed (Table 2). Unlike previous studies, we analyzed the global gene expression response to Mitsui7 in lung tissue and cells using comprehensive and high-content DNA microarrays to globally characterize potential mechanistic differences in response. Three major concepts emerged from this analysis: (1) the primary toxic response *in vivo* was an early acute phase and inflammation dose-response which was not activated *in vitro*; (2) the *in vitro* model is less responsive than the *in vivo* at lower doses and does not show a dose-response pattern in the induction of toxicity pathways at increasingly higher concentrations; and (3) both models respond by activating similar core cellular functions (oxidative stress and fibrosis) at the pathway level, however, regulation of most individual genes, the underlying mechanisms and consequent nature of the biological response are different. We suggest that careful consideration should be given to the *in vitro* concentrations selected, the types of cells used, and the endpoints studied when deriving regulatory decisions for ENM such as Mitsui7.

## Supporting Information

File S1 **Characterization of Mitsui7 in dispersion medium.**
(PDF)

Figure S1 **DLS measurements of Mitsui7 in different dispersion medium.**
(PDF)

Table S1 **Genes differentially expressed in vivo.**
(XLS)

Table S2 **Genes differentially expressed in vitro.**
(XLS)

Table S3 **Genes commonly enriched in the three dose groups in vitro.**
(XLS)

Table S4 **List of genes showing differential expression in control lung epithelial cells vs control lung tissue samples.**
(XLS)

Table S5 **List of genes validated by RT-PCR in lung tissue and in lung epithelial cells.**
(XLS)
