# Supplementary material for: Transcriptomic Analysis Reveals Novel Mechanistic Insight into Murine Biological Responses to Multi-Walled Carbon Nanotubes in Lungs and Cultured Lung Epithelial Cells
Source: PLoS One. 2013 Nov 19;8(11):e80452. doi: 10.1371/journal.pone.0080452 (PMC3834097; doi:10.1371/journal.pone.0080452)
Supplement: File S1 — Characterization of Mitsui7 in dispersion medium. (PDF) [file pone.0080452.s001.pdf]

Materials and Methods

Characterization of Mitsui7 in dispersion medium

Dynamic Light Scattering (DLS)

The average size of Mitsui7 in the dispersion medium at dose 162 µg used for *in vivo* exposures (0.9% NaCl in MilliQ water with 10% v/v BAL) and for *in vitro* exposures in cell culture medium at dose 100 µg/ml was determined by Dynamic Light Scattering (DLS) using a Malvern Zetasizer Nano ZS (Malvern Instruments Nordic AB, MAL1040112, Greve, Denmark). Analyses were performed on both unfiltered as well as 3.1-µm-filtered (Cellulose Acetate; DISMIC1-25CS; Toyo Roshi Kaisha Ltd., Japan) samples and 0.2 µm filtered vehicle to avoid interference from coarse particle agglomerates. Samples were measured at 25°C in 1 mL Malvern disposable polystyrene cuvettes. Data were analyzed using the Dispersion Technology Software version 6.2 (Malvern Instruments Nordic AB, Greve, Denmark). Analysis of hydrodynamic size was conducted through the refractive (R_i) and absorption indices (R_{abs}) of 2.020 and 2.000, respectively, for Mitsui7 and viscosity properties for H_2O (0.89 cP). For analysis of the *in vivo* vehicle we used the Malvern standard conditions for both water and protein (R_i = 1.450; R_{abs} = 0.001).
