# Supplementary material for: Transcriptomic Analysis Reveals Novel Mechanistic Insight into Murine Biological Responses to Multi-Walled Carbon Nanotubes in Lungs and Cultured Lung Epithelial Cells
Source: PLoS One. 2013 Nov 19;8(11):e80452. doi: 10.1371/journal.pone.0080452 (PMC3834097; doi:10.1371/journal.pone.0080452)
Supplement: Figure S1 — DLS measurements of Mitsui7 in different dispersion medium. (PDF) [file pone.0080452.s002.pdf]

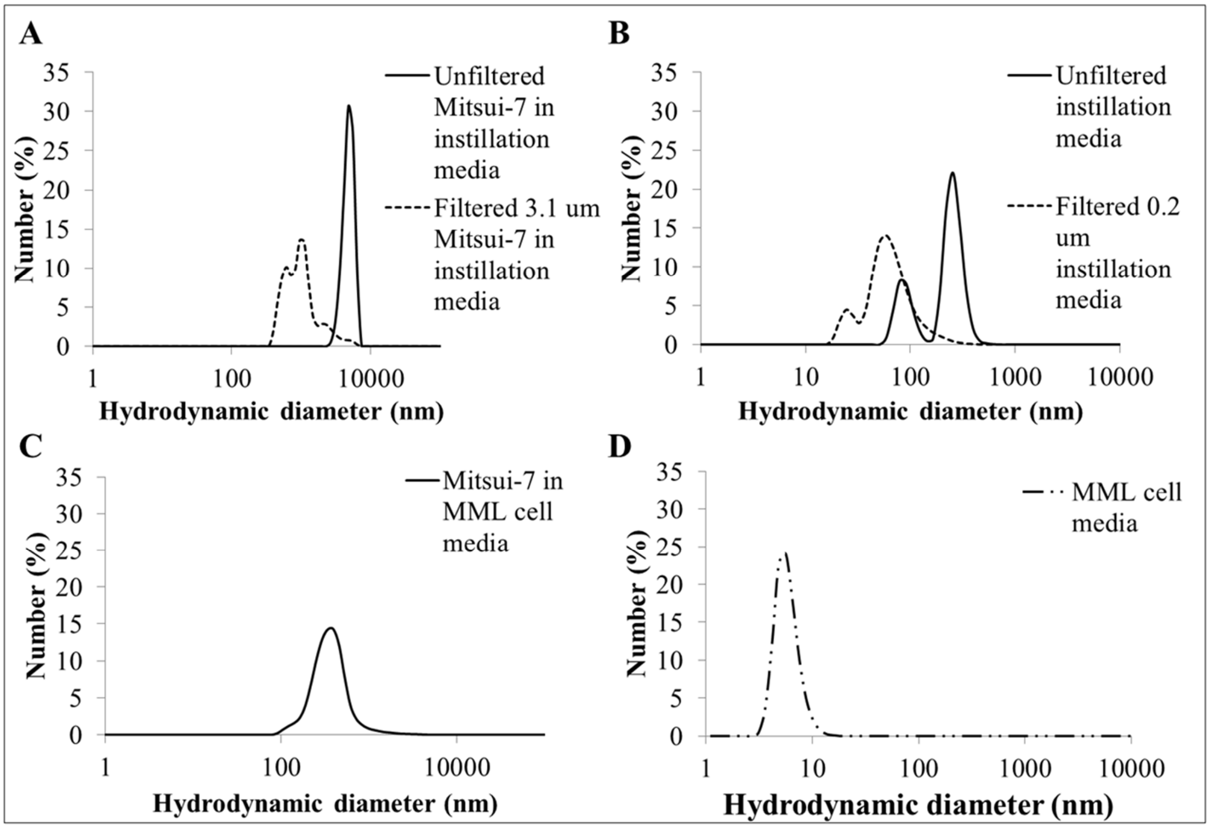

Figure S1: DLS measurements of Mitsui7 in different dispersion medium. (A) The hydrodynamic diameter (Dh) in nm of Mitsui7 in the *in vivo* exposure medium (MilliQ H_2O + 0.9% NaCl and 10% v/v acellular BAL). Solid line - unfiltered, dashed line - filtered 0.2 µm. (B) Dh of nonspecific (proteins) molecules in the *in vivo* exposure medium. Solid line - unfiltered, dashed line - filtered 0.2 µm. (C) Dh of Mitsui7 in unfiltered *in vitro* exposure medium (DMEM/F-12 + GlutaMaxTM-1 with 2% FBS, 1% pen/strep solution and 100 ng/µl murine EGF). (D) Dh of nonspecific (proteins) molecules in the *in vitro* exposure medium.
